# A generalized lineage nomenclature for viral genomic epidemiology

**DOI:** 10.1038/s41564-026-02468-3

**Published:** 2026-09-30

**Authors:** Rachel Colquhoun, Angie S. Hinrichs, Verity Hill, Christopher Ruis, Cornelius Roemer, Oliver G. Pybus, Andrew Rambaut, Áine O’Toole

**Affiliations:** 1 Institute of Ecology and Evolution, University of Edinburgh, Edinburgh, UK; 2 University of California, Santa Cruz, CA USA; 3 Department of Microbiology, Immunology and Transplantation, Rega Institute, KU Leuven, Leuven, Belgium; 4 Victor Phillip Dahdaleh Heart and Lung Research Institute, University of Cambridge, Cambridge, UK; 5 Department of Veterinary Medicine, University of Cambridge, Cambridge, UK; 6 Cambridge Centre for AI in Medicine, University of Cambridge, Cambridge, UK; 7 Biozentrum, University of Basel, Basel, Switzerland; 8 Swiss Institute of Bioinformatics, Basel, Switzerland; 9 Royal Veterinary College, University of London, London, UK; 10 Department of Biology, University of Oxford, Oxford, UK

**Keywords:** Phylogeny, Viral evolution, Taxonomy

## Abstract

Pathogen genomics has become increasingly integrated into outbreak response, with recent virus epidemics better characterized by large numbers of viral genetic sequences. Effective international communication of viral diversity requires a scalable, adaptable nomenclature, and existing systems may not accommodate rapid sequence generation. The Pango lineage nomenclature system was designed to address these problems and has been applied to SARS-CoV-2 since early 2020. Here we generalize the underlying principles into a framework for virus surveillance. Lineages are defined as epidemiologically meaningful clusters on a phylogenetic tree, initiated from founding lineages and expanded across a phylogeny through a hierarchical system of alphanumeric sublineage names. Six years on from the inception of the Pango system, we discuss challenges and considerations for implementation. We present a Pango lineage framework for chikungunya virus, providing an alternative to the geography-based clade system and designating 21 lineages across 8 founding lineages. This generalization provides a foundation for future genomic surveillance of many viruses, including for emerging outbreaks and the surveillance of endemic pathogens.

## Abstract (web summary)

This study extends the Pango lineage nomenclature system beyond SARS-CoV-2, introducing a dynamic framework for tracking and communicating viral diversity, demonstrated through its application to CHIKV.

## Main

The last decade has seen an enormous increase in the generation of virus genome sequence data and characterized virus diversity, and virus genomics has become a core pillar of the surveillance of both endemic and emergent viruses. Systems of virus nomenclature and classification are required to organize and make sense of this genomic information. The International Committee on Taxonomy of Viruses classifies viruses down to the species rank^1^. Description of viral diversity below the species rank has been historically undertaken by the scientific community, and virus variation has been subdivided using a wide variety of terms including *type*, *subtype*, *genotype*, *group* and *clade* (Extended Data Table 1)^2–4^. These naming systems typically categorize virus genetic diversity using a phylogenetic tree that is split into a set of *clades* (monophyletic or paraphyletic groups). The clades are often approximately equally divergent and well supported, thereby capturing persistent groups and enabling robust classification on a scale of months or years. However, heterogeneity among virus species in genetic diversity, in the evolutionary processes driving that diversity and in the availability of genomic data has led to a proliferation of classification systems across viral families. Many such systems were designed when far fewer viral genomes were available and may not provide sufficient granularity with the current pace and scale of virus genome generation.

Genomic sequencing during the COVID-19 pandemic has yielded to date more than 17 million publicly shared virus genomes and supported global real-time tracking of the virus’ evolution and spread. Public health actions during the pandemic were directly informed by virus genomics, and a critical enabler of this was the use of shared systems for naming virus genetic diversity, which facilitated unambiguous communication about virus clusters and variants worldwide. In 2020, Rambaut et al. proposed a dynamic nomenclature for SARS-CoV-2 lineages, called Pango, which was designed as a real-time framework for categorizing virus genetic diversity at a high level of phylogenetic resolution^5^. Over the subsequent years, a voluntary community has curated thousands of Pango lineages from the >17 million SARS-CoV-2 genome sequences, with the aim of capturing and characterizing the leading edge of the pandemic and achieving a globally consistent horizon scanning framework^6,7^.

The widespread adoption of the Pango lineage nomenclature for SARS-CoV-2, coupled with substantial increases in global sequencing capacity as a result of the pandemic, has led to the development of similar lineage systems for other viruses, including mpox virus (MPXV)^8^, rabies virus^9^, human respiratory syncytial virus^10^ and dengue virus^11^. As genomics becomes a fully integrated component of viral outbreak response and surveillance, many other viruses will require a fine-scale nomenclature capable of categorizing epidemiologically relevant diversity^12^. Here we outline the key principles of a unified dynamic virus lineage system that formalizes and generalizes the Pango lineage nomenclature. We highlight the applications that are most amenable to a Pango-style nomenclature framework and offer practical guidance for implementation. We present this framework with genomic epidemiology in mind, although Pango lineages could also be applied to well-sampled non-human outbreaks in the case of epizootics.

## Results

### Principles of a Pango lineage system nomenclature

A Pango lineage nomenclature aims to capture the epidemiologically relevant diversity of a given pathogen on the phylogenetic scale that is most informative to address public health questions. Any such system should therefore be established with a clear public health or evolutionary objective in mind—such as informing control or eradication efforts, tracking outbreak transmission dynamics or monitoring viral evolution in response to new vaccines or therapeutics. By providing a common language for describing emerging viral clusters, a nomenclature system enables consistent communication across institutions, regions and national borders. It can serve as a practical approach to the phylogenetic placement of newly generated virus genomes when datasets are exceptionally large or when users lack the computational resources or time to reconstruct phylogenetic trees de novo, thus enabling rapid data interpretation and decision-making.

**Fig. 1:**
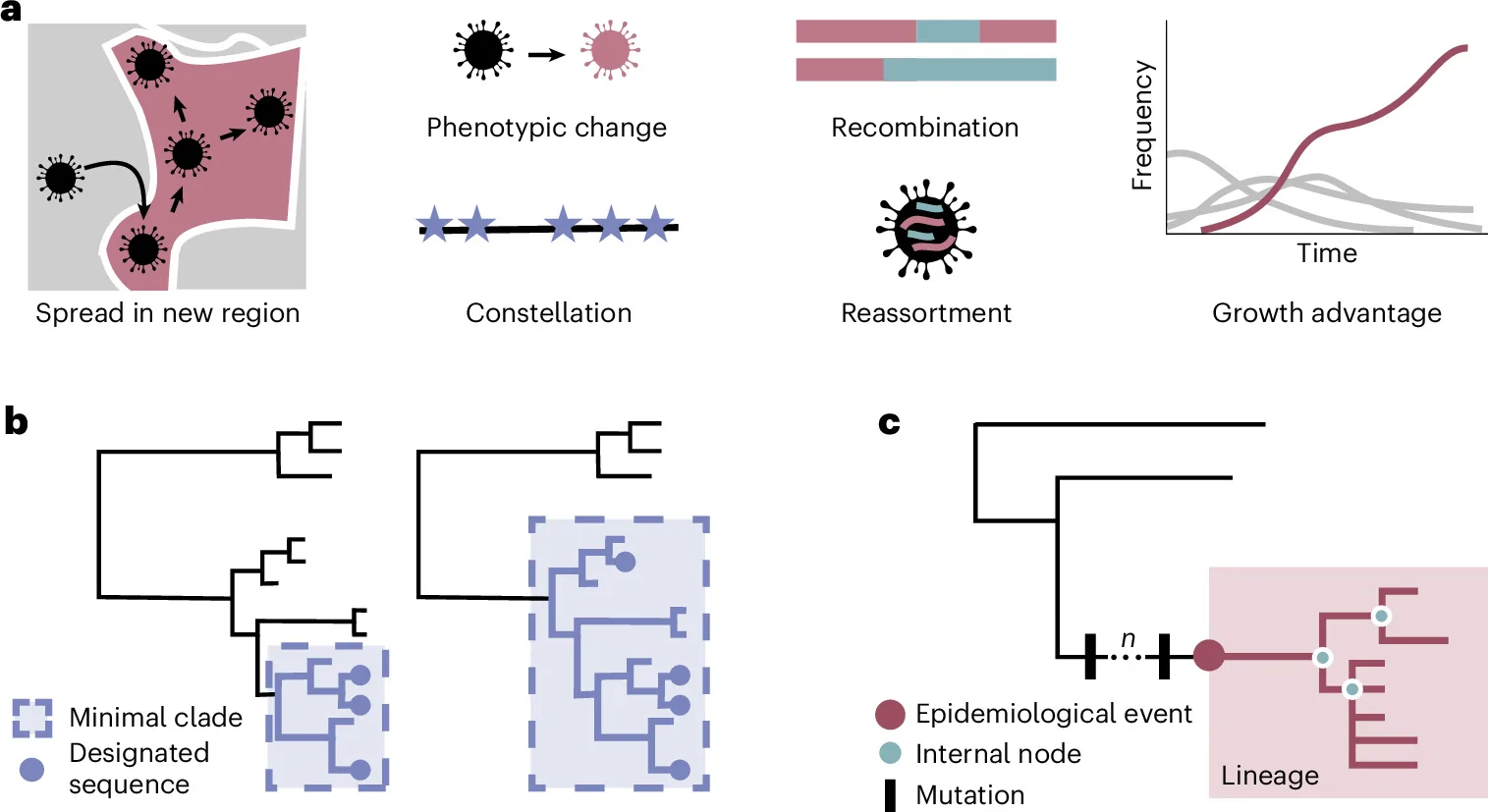
Definition of a Pango lineage. **a**, Lineages are *designated* when phylogenetic clusters show epidemiological relevance, including introduction into a new region with evidence of onward spread, a change in phenotype, a notable constellation of mutations, a recombination or reassortment event, or rapid relative growth (see main text). **b**, A lineage is defined as the minimal clade containing the set of all virus sequences descending from a most recent common ancestor, defined by the set of sequences designated to that lineage. Here two examples illustrate how the lineage scope (dotted line) is determined by the set of designated sequences (blue dots). **c**, A lineage aims to capture an epidemiologically relevant event and all of its descendant sequences. In order to be a new lineage, it must carry one or more mutations distinguishing it from the parent diversity and show evidence of onward transmission in the form of internal nodes.

A Pango lineage is a set of virus sequences that form an epidemiologically relevant cluster on a phylogenetic tree. The data in question can represent either complete genomes or partial genome sequences; however, greater granularity will be achieved if complete genomes are used^13^. A range of signals may justify the definition of a new lineage, depending on the epidemiology, evolution and socio-medical context of the virus in question. Possible signals include (1) introduction into a new region with onward transmission, (2) changes in phenotype, (3) a notable set (constellation) of mutations that may have phenotypic relevance, (4) recombination or reassortment or (5) evidence of rapid growth relative to other lineages (Fig. 1a). Depending on the virus and its context, only some of these, or distinct, signals may be relevant. For SARS-CoV-2, early lineage definitions were informed by geographical clustering, as movement of people was limited owing to travel restrictions^14,15^. As the pandemic progressed and global travel resumed, lineage criteria shifted to other epidemiologically relevant signals, including growth advantage, recombination and mutations associated with possible immune escape or vaccine evasion^16–19^.

In the Pango lineage system, sets of sequences and a phylogenetic tree are used to define which sequences belong to which lineage (Fig. 1b). The system discriminates between two different modes of categorization: *designation* and *assignment*. In the *designation* process, a set of *designated* sequences are defined, which represent a curated subset of high-quality sequences that definitively and unambiguously belong to that lineage. Thus, the designated sequences form a canonical reference set for a lineage within a given Pango lineage system (although summary information, such as defining mutations, may also accompany this definition). For any given phylogenetic tree, the designated sequences define the scope of the phylogenetic clade of the given lineage. In some cases, the designated sequences will comprise all the genomes within a lineage; however, in many cases, the designated sequences will comprise only a fraction of the total lineage. This will depend on the rate of data generation. When data generation is slow, all high-quality sequences can be feasibly designated. However, for very large or rapidly changing datasets, only a small fraction of new genomes will be designated, with the remainder classified through assignment tools (as was the case for SARS-CoV-2).

Sequences that are not designated to a lineage can still be classified through a different process called *assignment*. Assignment is performed by one or more of a range of methods, including traditional phylogenetics or time-saving approaches such as phylogenetic placement, assessment of defining mutations or machine learning-based classifiers^7,20,21^. The assignment process is an inferential procedure and therefore carries some uncertainty; it is possible (although unwelcome) for different assignment methods to give different classification results, and this uncertainty should be accounted for when interpreting the results. In addition, assignment may be performed on lower-quality or incomplete sequences, whereas only high-quality sequences are suitable for designation.

### Definition of a Pango lineage

A lineage is defined formally as all viruses that subtend from the most recent common ancestor defined by the set of designated sequences (Fig. 1b). A new lineage must meet the following criteria (Fig. 1c):

1. It is genetically distinct from the ancestral lineage by one or more mutations.
2. It has epidemiological relevance, as assessed by a previously defined set of relevant signals.
3. There is evidence of onward community transmission.
4. There are a sufficient number of high-quality genomes to evaluate these criteria.

The exact thresholds for these criteria will necessarily differ among viruses owing to differences in evolutionary rates, sequencing intensity, the length of time the virus has been circulating in humans and public health use cases. For example, the number of mutations that separate a new lineage from its ancestral diversity may be lower in slower-evolving viruses, or when fine-scale resolution is needed, and the number of genomes required to define a lineage may depend on the volume of data available for a given outbreak. Specific criteria and thresholds should be set for each virus or use case, but can be applied flexibly to allow for unforeseen circumstances, and may also change through time as the epidemiological context changes.

### A generalized framework for Pango lineages

**Fig. 2:**
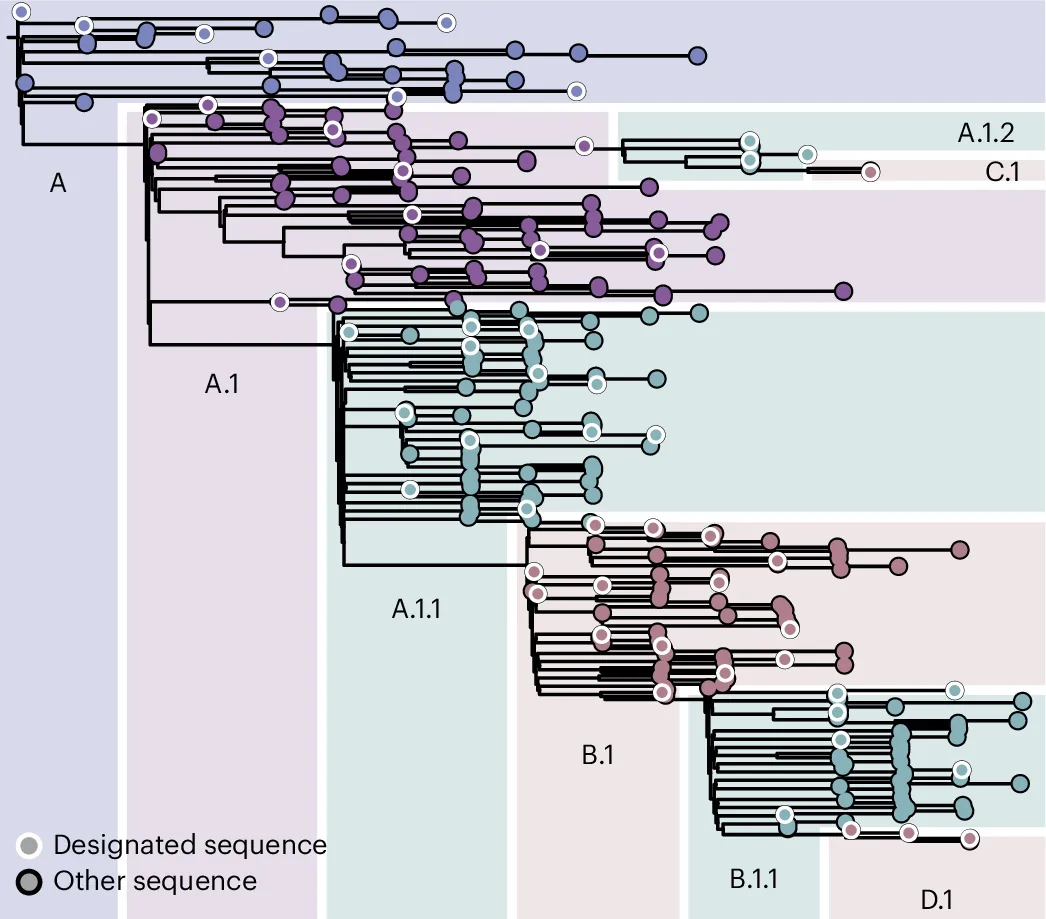
Lineages have a phylogenetically defined hierarchy. In the Pango lineage nomenclature, a phylogeny is partitioned hierarchically into *lineages*. The hierarchical levels represent ancestry and descent. Lineage names are alphanumeric, with letters representing a given hierarchical level and numbers representing the order of designation at that level. Therefore, A.1 is a descendant sublineage of lineage A. The first A.1 sublineage is labelled A.1.1, the second is labelled A.1.2, and so on. At a defined hierarchical level, an alias is introduced in order to shorten the lineage name and maintain readability; however, this alias must be recorded and can be expanded back out to reflect the full ancestry of the lineage. Here the alias is introduced after two numeric sublevels so that B is an alias of A.1.1. The first sublineage of A.1.1 takes on the alias as B.1, and so on. A high-quality representative subset of sequences is designated to define a given lineage.

**Fig. 3:**
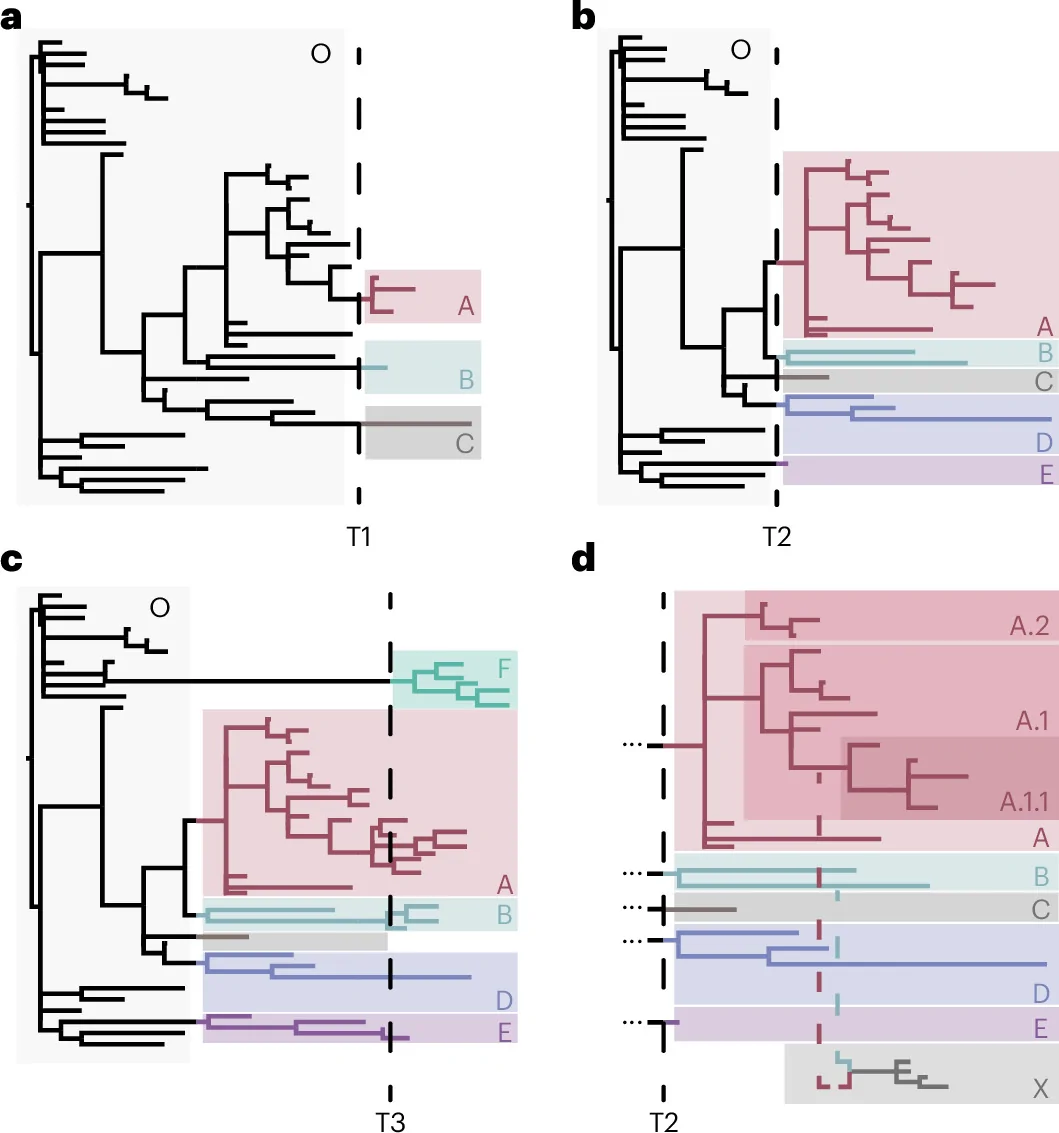
How to begin and revise a Pango lineage nomenclature system. **a**, Choice of initial time point results in differing numbers of founding lineages to capture the circulating diversity, and sequences existing before this cut-off are labelled ‘O’. At time point T1, there are three founding lineages circulating, named A, B and C. **b**, Selecting an earlier time point T2 results in five founding lineages (A to E). The initial time point selection should be informed by what diversity is relevant to capture for public health. **c**, If, at a later time point, T3, historic diversity that had been classified as ‘O’ again becomes epidemiologically relevant, it can be incorporated into the system with the next available alphabetic prefix (F). If some previously classified diversity is no longer circulating, those lineages can be dropped from the system (lineage C). **d**, Following the founding lineages, relevant clusters can be labelled as sublineages (for example, A.1, A.2 and A.1.1). A circulating recombinant can be added to the framework using the next available X-prefixed alphabetic code. Here parent lineages A.1 and B give rise to a recombinant, X. The ancestry of the recombinant lineage is stored as metadata, and any sublineages of X will be labelled X.1, X.2 and so on.

A Pango lineage system provides a *hierarchical partition* of a phylogenetic tree for a given virus, with the explicit goal of supporting genomic epidemiology for public health (Fig. 2). A lineage system is anchored on a set of *founding lineages* present at an initial time point of interest. For SARS-CoV-2, the earliest sequenced genomes were grouped into two founding lineages, A and B, distinguished by two mutations, which were later found to correspond to a scenario of at least two separate introductions of the virus into the human population^22^. For the sustained human outbreak of clade IIb MPXV, a single founding lineage, A, captured the initial diversity^5,8^. For any Pango lineage system, we recommend choosing an initial time point to divide existing viral diversity into one or more founding lineages, each labelled with an alphabetic code on a phylogeny (Fig. 3a,b).

In the case of zoonotic spillover giving rise to a human outbreak, it would be intuitive to use the point of spillover as the initial time point. For endemic viruses with pre-existing genetic diversity, the starting point should capture currently circulating diversity while excluding extinct lineages in order to minimize complexity and reduce the burden of characterizing historic data (Fig. 3). Nomenclature systems that are less granular and use more persistent lineage labels may be better suited to describing longer-term endemic diversity.

Genetic diversity that predates the chosen initial time point can be treated as ‘outside’ the nomenclature system (O). There is no requirement for Pango lineages to be similar in size, nor a need to designate all pre-existing diversity. Any undesignated diversity that later becomes relevant for public health can be added to the Pango lineage system as an additional founding lineage (Fig. 3c). Given the set of founding lineages, the designation of further descendant lineages is thereafter guided by the lineage designation rules and associated genotypic and epidemiological criteria (Fig. 3d). In situations where an established clade system already exists, the founding lineages for a Pango-style system may align directly with existing nomenclature labels, which may be particularly useful for re-emerging outbreaks of an endemic pathogen.

Lineage names follow a dot-separated alphanumeric structure that encodes local phylogenetic (ancestor–descendant) relationships (Fig. 2). A new sublineage is defined by appending a dot and the next available number (for example, the 75th sublineage of BA.2 to be designated becomes BA.2.75). The names themselves should not attempt to encode any other features, such as phenotypic information or specific mutations; these features can be stored in associated metadata entries for each lineage. To maintain lineage name readability as the virus diversifies, an alias is introduced at a defined hierarchical level. SARS-CoV-2 lineages adopt an alias after three levels, whereas the MPXV system introduces an alias following two. The alias replaces the entire prefix using the next available alphabetic code (excluding X, I and O, which are reserved letters). Once all single letters have been applied, aliases will be formed using two letters; these aliases continue to be incremented alphabetically with the right-hand-most letter incremented first. For example, AA is the first alias following Z, while AB immediately follows AA, and BA immediately follows AZ. Should aliases of greater length than two letters be required, their alphabetic incrementation begins with the right-hand-most letter and proceeds with letters from right to left.

When a circulating recombinant is identified, a new founding lineage is designated with the next alphabetic code prefixed with an X (Fig. 3d). For example, SARS-CoV-2 lineage XBB is a recombinant of BJ.1 (BA.2.10.1.1) and BA.2.75 (BA.2.75.3.1.1.1), and the next circulating recombinant identified and designated following XBB would be XBC, and so on. Knowing the number and exact location of precise recombination breakpoints is not required for designation, although they should be recorded where known in the associated lineage metadata entry. Note that designation can proceed even when there is uncertainty in the identification of the parental lineages of a recombinant lineage.

For reassorting viruses, in which virus genome segments follow distinct evolutionary ancestries, a Pango lineage nomenclature can be anchored to a single representative segment. The choice of segment can be adapted for the intended application; for example, an appropriate choice for genomic epidemiology will be the one most relevant for transmission or antigenicity, which is often the fastest evolving and can provide the most resolution over short timescales. Reassortment of the other segments that are epidemiologically relevant could trigger a new lineage designation, an event that could be specifically encoded in the phylogeny of the representative segment for assignment purposes. Broader clade nomenclatures can track the diversity of the other segments and inform the identification of reassortants^23,24^.

Because the goal of Pango is to capture the emerging edge of an outbreak, the phylogeny and lineage definitions must remain dynamic in response to the discovery of new information (Fig. 3c). Lineages can be withdrawn from use if, in the light of additional data or new epidemiological information, their designation criteria are no longer met (any such lineage names should be permanently deprecated and never reused). Lineages can also be split if a previously monophyletic group subsequently resolves into multiple epidemiologically relevant clusters or revised to include different sequences following new sampling. This flexibility allows early action, rather than waiting for stable clades to form through further ongoing transmission and evolution. For SARS-CoV-2 lineages, minor revisions were often needed to refine definitions as new data were shared; however, these definitions were available to the research community much more rapidly as a result of a willingness to act early and review them.

### Granularity of a Pango-style nomenclature

No single nomenclature can serve all purposes, and a Pango lineage system is not intended to supersede existing taxonomy. Rather, it functions as a complementary framework alongside naming schemes with different granularities and timeliness.

For endemic pathogen surveillance that largely samples deep, historic diversity, a fine-scale lineage nomenclature such as Pango may be of limited utility. Traditional phylogenetic analyses and clade-based nomenclatures, which incorporate minimum size and persistence criteria, are better suited to capturing long-term evolutionary patterns such as antigenic turnover^8,23^. In these contexts, a fine-scale system may introduce unnecessary complexity by overcategorizing the phylogenetic diversity without a clear benefit to public health. However, a Pango lineage system may be valuable for endemic pathogens in cases when new selective pressures are expected to shape short-term evolution, such as following the introduction of a novel vaccine or antiviral treatment (for example, dengue virus or respiratory syncytial virus), or when eradication programmes require close tracking of contemporary transmission (for example, measles virus or poliovirus).

A Pango approach is especially well suited for outbreaks following a zoonotic spillover, for which emerging pathogen diversity should be tracked in real time. However, to justify a Pango nomenclature and lineage system, pathogens will need to accumulate sufficient genetic diversity during the outbreak, often referred to as ‘measurably evolving’^25,26^. Many RNA viruses evolve sufficiently rapidly to provide meaningful information over the timescale of an outbreak; however, some DNA viruses, such as double-stranded DNA viruses, evolve too slowly to be informative, unless host-mediated mechanisms—such as APOBEC3 editing—drive virus mutation rates higher^27^. Viruses that do evolve sufficiently quickly may not always be suited to a lineage system or may have different lineage profiles. For instance, influenza A virus has a high turnover of lineages, with many extinct lineages, whereas HIV-1 has a stable set of genetically diverse strains that continuously co-circulate and recombine. A system of recombinant Pango lineages may be possible for HIV-1; however, the current nomenclature for HIV-1 circulating recombinant forms is functioning well for existing use cases^2^ and does not need to be replaced. It may also be possible to apply the Pango lineage framework to pathogens with larger genomes, such as bacteria, although the added complexity of horizontal gene transfer would need to be carefully considered. In such contexts, public health priorities such as antimicrobial resistance tracking should be central to a bacterial lineage system.

**Fig. 4:**
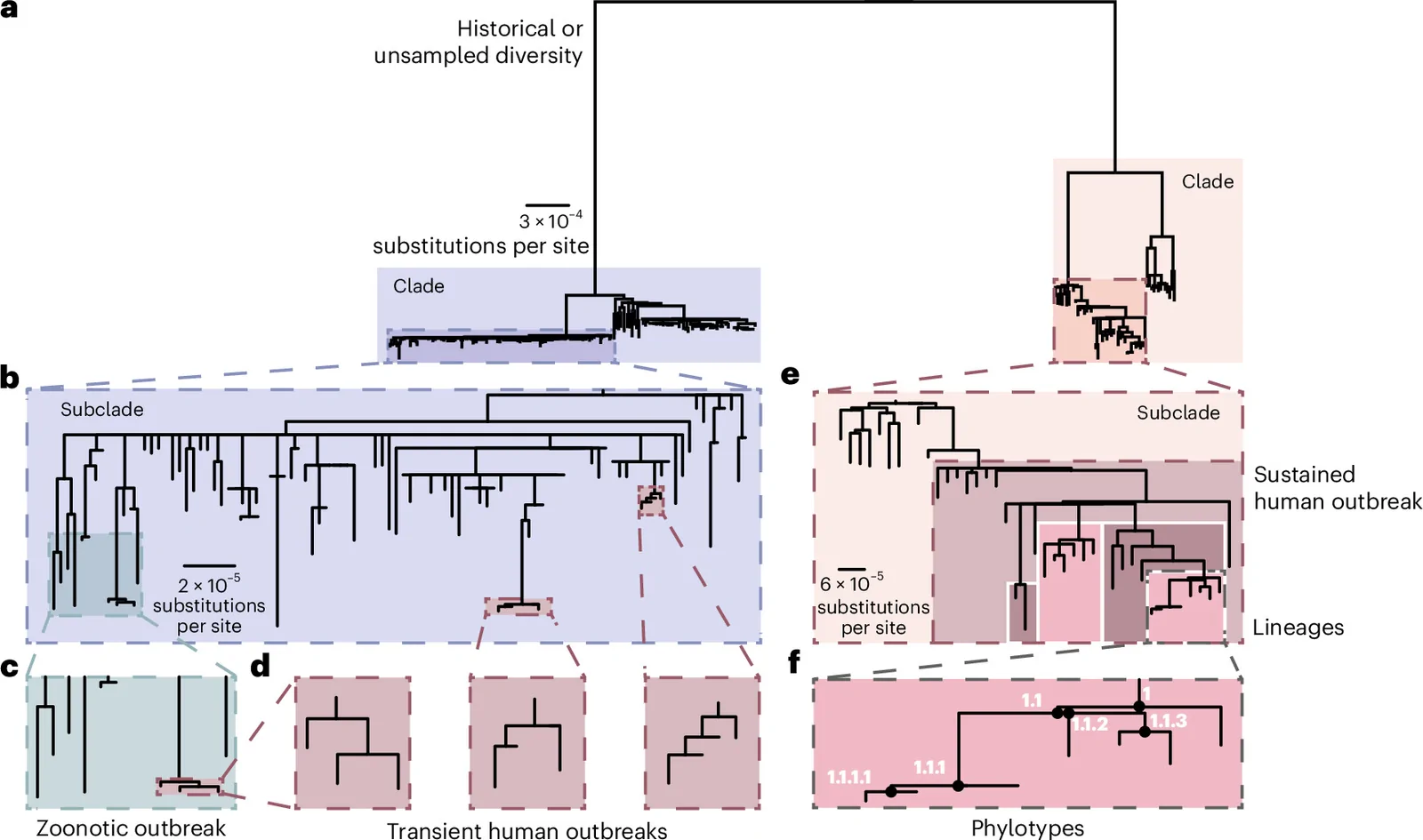
Pango lineages can exist alongside other terms that fulfil distinct purposes. **a**, To characterize the deep ancestry of a pathogen, particularly one with largely unsampled reservoir diversity, a persistent clade system will be useful. **b**, When necessary, *clades* can be split into *subclades*, or *groups*, that describe finer-scale relationships. **c**, Human outbreaks resulting from an increase in the rate of zoonotic cross-species transmission of genetically distinct viruses can be described as *zoonotic* outbreaks. **d**, Self-limiting transmission chains following a spillover event are classed as *transient human* outbreaks. Any given zoonotic outbreak may give rise to a transient human outbreak. **e**, If the outbreak is not self-limiting and is detected over a prolonged period in multiple locations, the outbreak is a *sustained human* outbreak, and a Pango *lineage* system may be useful for tracking the outbreak as it unfolds. **f**, For local investigations at the highest level of phylogenetic resolution, *phylotypes* encode all nodes in a phylogeny to provide an intuitive sense of relatedness for those not familiar with how to read a tree.

Furthermore, not all types of outbreak or epidemic will require or benefit from a lineage system (Fig. 4). *Zoonotic outbreaks*, in which rises in cases are due to elevated levels of zoonotic spillover—such as the 2018 Lassa fever outbreak in Nigeria due to many independent spillovers from the *Mastomys* reservoir^28^ and re-emergences of yellow fever virus in Brazil^29^—do not require a lineage system. Similarly, a zoonotic event that results in a self-limiting, geographically confined human transmission chain (*transient human outbreak*) will not typically warrant a lineage system, as the outbreak does not persist long enough for it to be necessary or useful.

Dynamic lineage systems are most valuable for outbreaks that exhibit sustained human-to-human transmission across multiple regions. For example, the clade IIb MPXV outbreak that spread globally in 2022 illustrates how a Pango lineage system can be used alongside and complement existing *clade* and *outbreak* nomenclatures^8,30^. In the context of a *sustained human outbreak*, a lineage nomenclature system provides a shared language that supports rapid public health response and cross-agency communication. Independent spillover events that give rise to separate sustained human outbreaks would typically warrant independent lineage systems. For example, MPXV clade IIb sh2017 has a Pango-style lineage system; however, if another independent spillover event gave rise to a novel sustained human outbreak, this would initiate a new Pango lineage system for that outbreak^30^. A Pango lineage system is directly applicable to enzootic pathogens and can be used to characterize and track viral diversity in animals other than humans. In the case of the rabies virus lineage nomenclature, the lineage system broadly captures diversity circulating in dogs, with humans as dead-end hosts^9^. Furthermore, if multi-host dynamics are central to surveillance needs, the framework can include these events as part of lineage designation.

A key advantage of a fine-scale, dynamic system such as Pango is its responsiveness; however, there is a balance between defining too few and too many lineages. Designating every node on a phylogeny as a separate lineage produces clusters that are genetically similar, reducing classification accuracy, increasing phylogenetic uncertainty and obscuring rather than highlighting meaningful patterns of viral spread. Conversely, defining too few lineages risks losing the resolution needed to track epidemiologically relevant clusters. In some cases, phylogenetic resolution that is even finer than lineages may be needed, for example, for detailed local or regional investigations, such as nosocomial outbreaks in a hospital or across a care network^15,31^. To facilitate reporting genomic results without requiring phylogenetic expertise, the term *phylotype* describes a dot-separated number notation encoding phylogenetic relationships across an entire phylogeny. Pango lineages occupy an intermediate level of granularity between monitoring long-term trends with clade nomenclature and exhaustive node-level classification with phylotypes (Fig. 4).

### Real-time lineage implementation

To be practical and effective, lineage nomenclatures must identify emerging diversity rapidly, reproducibly and in an unbiased way without extensive reliance on manual work by volunteers. Several efforts have been made towards an automated lineage designation process^9,32^; however, current approaches are limited by the complex genomic and epidemiological landscape of outbreaks and still require manual curation and review of lineages by a volunteer community consisting largely of academics.

A Pango lineage system is designed explicitly to support public health, and in a highly interconnected world, outbreak surveillance must be globally inclusive and relevant. However, the scale of global genomic surveillance is very heterogeneous: high-income countries are overrepresented in public databases, whereas many regions contribute few sequences despite their epidemiological importance^33^. Lineage designation systems must therefore account for this disparity by applying stricter thresholds in well-sampled countries and giving greater weight to sequences from underrepresented regions. Crucially, those for whom the local data and lineages are most relevant should be empowered to contribute to, and flag new lineages within, the global nomenclature. This requires a transparent, public forum for proposals and review. Platforms such as GitHub provide an open, free and shared space for lineage hosting, helping democratize and share the maintenance burden.

### Lessons from SARS-CoV-2

The COVID-19 pandemic demonstrated that phylogeny-based nomenclatures can enable tracking of emerging variants and lineages in near real time. The Pango lineage nomenclature system for SARS-CoV-2 facilitated the rapid identification of lineages with growth advantages or phenotypically relevant mutations, allowing the scientific community to evaluate and monitor emerging variants as they arose. However, it soon became apparent that the Pango labels were not well suited for communication with the general public, necessitating the simpler, single-word WHO Greek-letter system (for example, Alpha, Beta and Gamma) for variants of concern or interest^19^. Despite the introduction of the WHO nomenclature, after Omicron, no additional Greek-letter variants were described, and the WHO has continued to use Pango lineages for tracking novel variants of interest and concern up to the present.

The global scale of viral genome production during the pandemic quickly surpassed the assumptions made when the Pango lineage system was initially developed. Before the pandemic, few researchers would have predicted that more than 100,000 genomes would be generated; the total currently stands at more than 17 million. Rambaut et al.^5^ anticipated that no more than approximately 200 active lineages would be necessary and that ‘any more would obfuscate rather than clarify discussion’. In practice, there were typically between 200 and 1,000 distinct lineages observed in any given month throughout much of the pandemic. In part, the number of lineages reflected the extraordinary diversity and scale of genomic surveillance and public data sharing, offering an unexpected level of detail as the virus evolved. In addition, advances in large-scale phylogenetics^34–36^ and the development of platforms such as cov-spectrum.org, cov-lineages.org, outbreak.info and covariants.org^37–39^ enabled users to more easily catalogue and track a large number of lineages. Likewise, tools such as Nextclade and Pangolin made lineage assignment for new data a trivial issue^7,20^. Although the pace and scale of lineage designations increased the volume of work necessary to maintain the Pango lineage system, novel tool development and community engagement offset this challenge and made implementation possible.

### A dynamic and time-limited nomenclature

The Pango lineage nomenclature was conceived as a dynamic and time-limited system; it was expected to be ‘most useful for the duration of the global pandemic’ rather than for long-term use^5^. In the wake of the acute pandemic phase of SARS-CoV-2, surviving lineages can retain their Pango names in a less granular and less responsive framework. More generally, when an epidemic pathogen transitions to endemicity, a more stable, persistent nomenclature system with a lower burden of maintenance may be better suited to long-term surveillance. A Pango lineage nomenclature should exist only for as long as it retains clear utility and support. Future consideration should be given to how outbreak nomenclature systems should be supported; at present, there are multiple beneficiaries of such systems (for example, public health agencies, public and private research centres, universities and industry), but no structure or organization exists to fund their establishment and maintenance. A sustainable model for lineage system maintenance should leverage existing distributed expertise across public health laboratories and academia, coordinated through a central governance framework and modelled on established pathogen surveillance networks^40,41^.

**Fig. 5:**
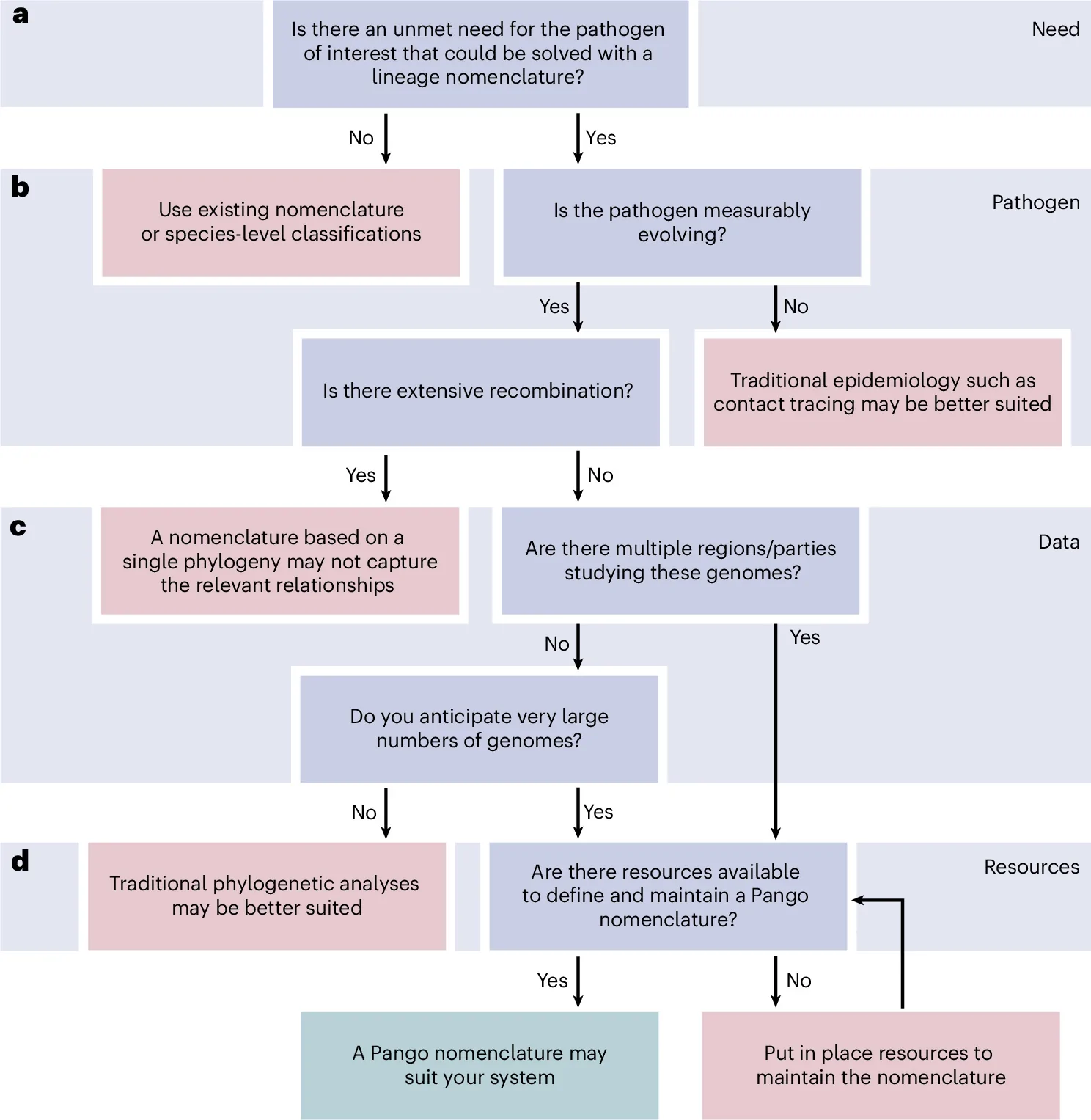
Guidelines for when a Pango lineage nomenclature system should be established. **a**, There is overhead in creating and maintaining a lineage nomenclature, and not every pathogen system is suited for it, or even needs it. If there is a challenge in the field that existing nomenclature is not sufficiently addressing, a lineage nomenclature may be of benefit. **b**, The nature of the pathogen of interest is important. Is it evolving sufficiently quickly that it accumulates diversity across the timescale of interest? If not, a fine-scale nomenclature may not be informative for public health, as epidemiological events may occur faster than detectable genetic changes. Furthermore, if the virus has a lot of standing diversity and recombines extensively, it may not be suited to a phylogeny-based nomenclature. **c**, If there are many partners investigating the data, and if there is an extensive amount of sequence data, a lineage system can help with communication and reporting. If very small numbers of genomes are anticipated, traditional phylogenetic methods may be sufficient to handle the diversity, and communication may be less challenging. **d**, A static nomenclature is of little use to public health during an emerging outbreak. Before implementing a system, there must be resources in place to maintain such a nomenclature. Once maintenance plans have been established, a Pango lineage nomenclature can be initiated.

Before implementing any new lineage nomenclature, users should critically evaluate whether a phylogenetic lineage nomenclature is fit for purpose. For some applications, a mutation-based or antigenic nomenclature, or more persistent clade nomenclature would provide more interpretable or durable frameworks (Fig. 5). A Pango lineage system can be a powerful tool for tracking emerging variation in real time, but should not be adopted in all cases.

### A Pango lineage system for chikungunya virus

**Fig. 6:**
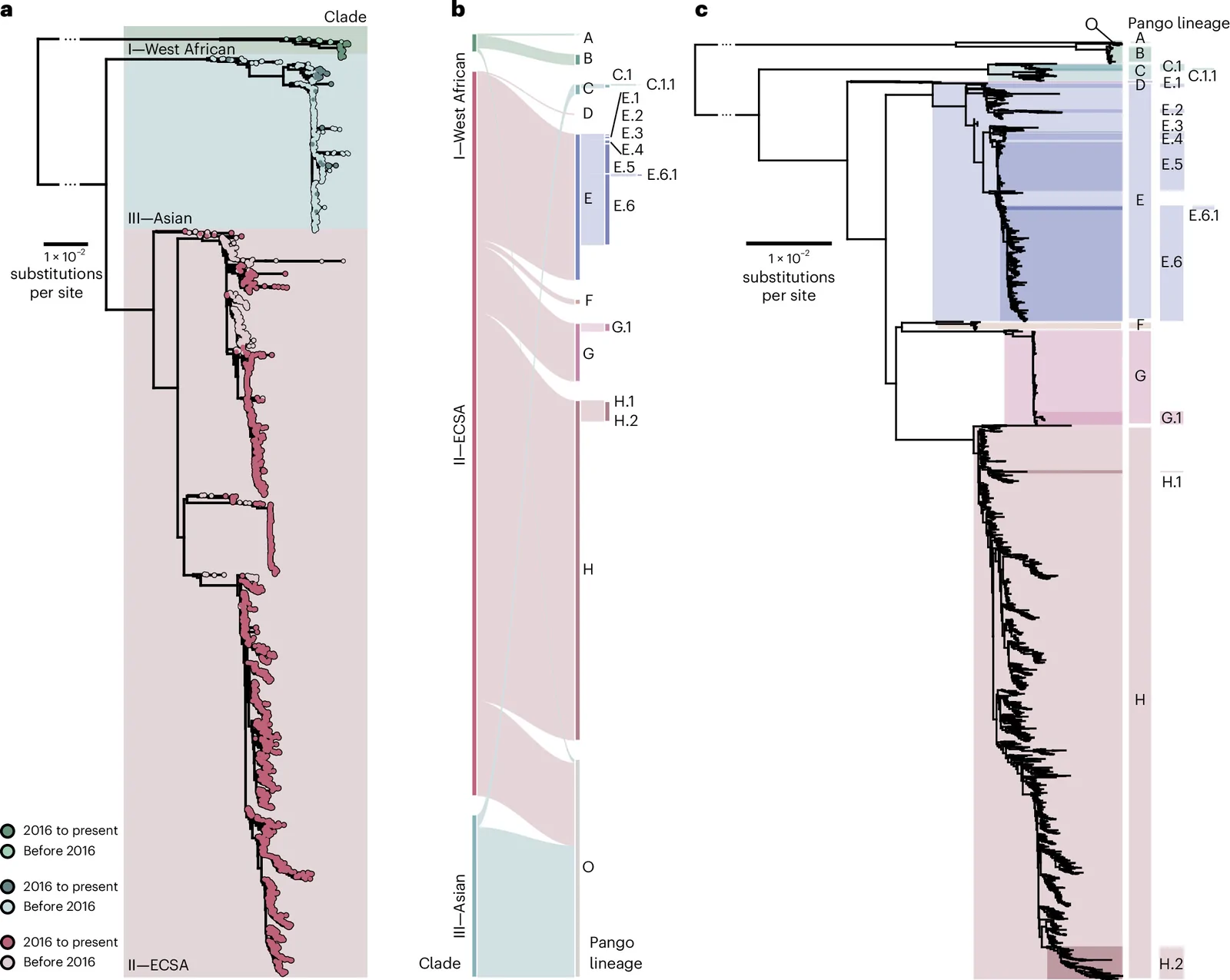
The Pango lineage nomenclature system applied to CHIKV. **a**, The current clade nomenclature for CHIKV splits all known diversity of the virus into three clades (I—West African, II—ECSA and III—Asian). Diversity from the last 10 years (2016 onwards) is highlighted within each of the clades. **b**, The CHIKV Pango lineage system subdivides recent CHIKV diversity into 20 lineages: A–H as founding lineages, with a number of hierarchical sublineages designated. Diversity sampled before 2016 is designated O. **c**, Pango lineages were annotated on a maximum likelihood phylogeny of publicly available diversity sampled since 2016. A CHIKV genome sequence sampled from a mosquito in China in 2021 is not closely related to other known diversity and falls outside of a lineage definition, designated O (accession number OQ054327).

**Extended Data Fig. 1:**
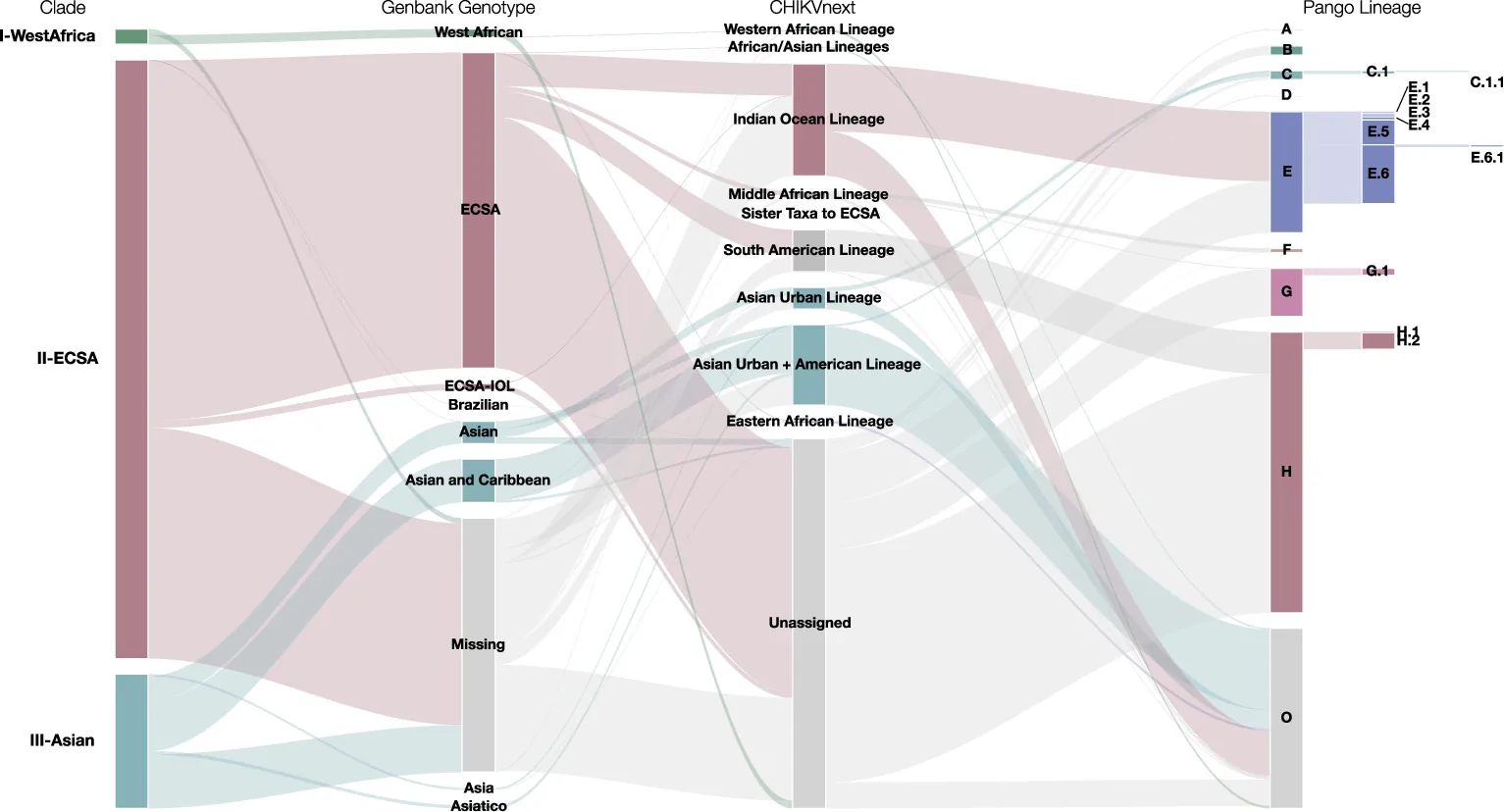
Mapping of existing CHIKV nomenclature to CHIKV Pango lineage. CHIKV clade annotations sourced from NextStrain, genotype annotations sourced from Genbank (with some post-hoc curation to harmonise to a common annotation), CHIKVnext lineage annotations from https://nextstrain.org/community/ViennaRNA/CHIKV, and the designated Pango Lineages. Many of the Genbank records lack a genotype annotation (marked as missing), and the CHIKVnext annotations are applied to virus samples taken up until 2022, more recent data is not hosted. Pango lineages focus on the most recent data (last 10 years), giving diversity that has not been seen for more than a decade the designation O.

Pango lineage nomenclatures have been applied to outbreaks following zoonotic events (for example, SARS-CoV-2 and MPXV). To provide a worked example of how a Pango nomenclature can be applied to a virus with a longer history of circulation in the human population, we developed a lineage system for chikungunya virus (CHIKV). CHIKV is a mosquito-borne alphavirus, first identified in 1952, and has since been reported in 114 countries^42,43^. Approximately 3,300 CHIKV genome sequences from more than 90 countries are publicly available on GenBank. CHIKV diversity is conventionally subdivided into three clades or genotypes: West African, East/Central/South African (ECSA) and Asian (Fig. 6a). Although finer subdivisions have been proposed (for example, Indian Ocean lineage^44^, Asian Urban + American lineage^45,46^ and ECSA-2^47^), naming conventions vary, and geographically defined labels do not reflect recent phylogeographic trends^45,46,48^ (Extended Data Fig. 1). In addition, the location-based nomenclature frequently lacks standardization (for example, East-Central-South-African^49^ versus East/Central/South African^48^; Extended Data Table 2).

**Extended Data Fig. 2:**
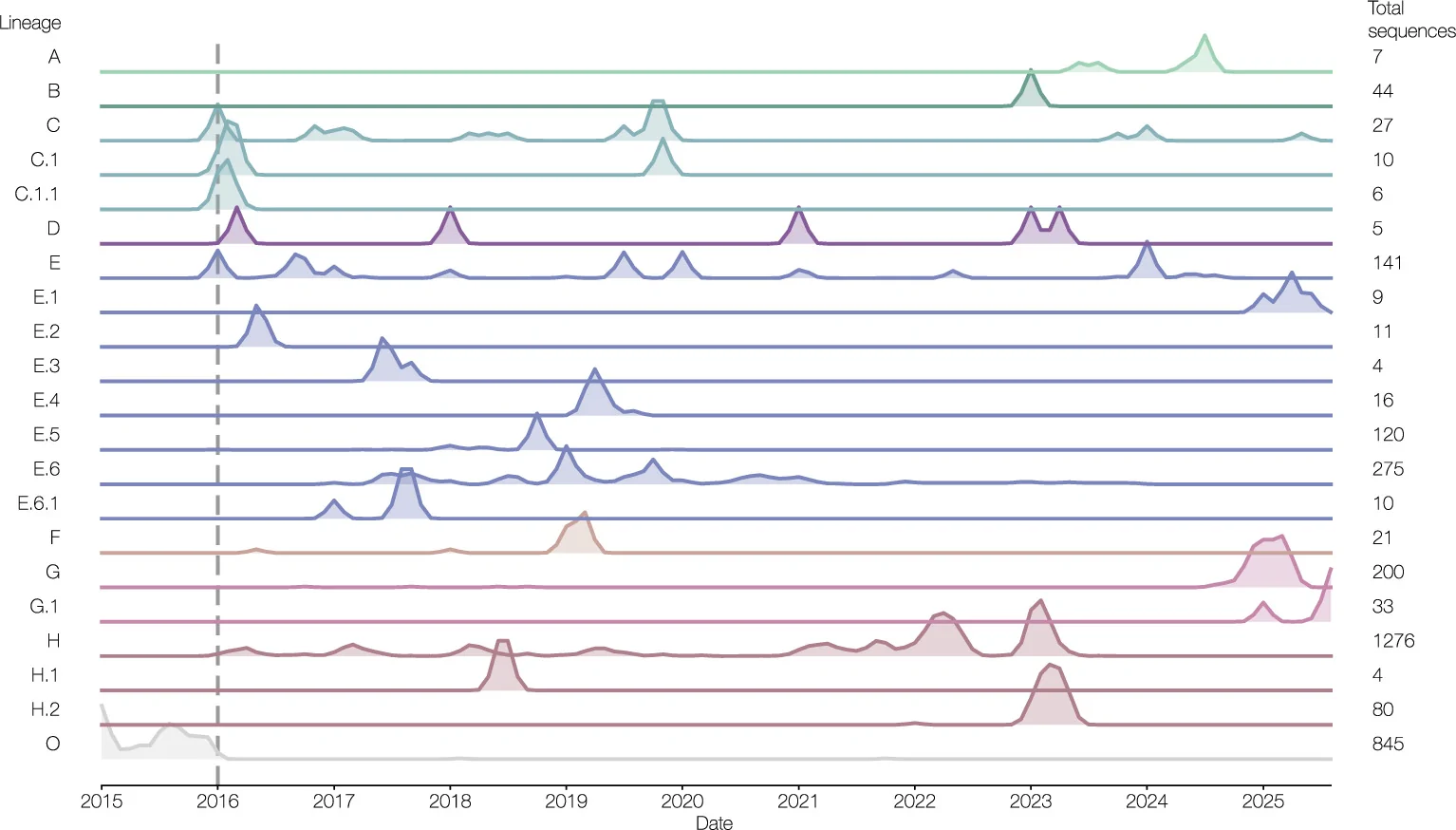
Temporal spread of CHIKV Pango lineages. The CHIKV Pango lineage system captures the sampled and sequenced diversity of CHIKV since 2016 in 21 different categories. Eight founding lineages are defined, with sublineages hierarchically descending from them. Any sequences sampled prior to 2016 are categorised as O, however if any O diversity is subsequently seen circulating in contemporary data, it can be added to the lineage scheme as an additional founding lineage.

We applied the generalized Pango principles to define an example CHIKV Pango lineage system. We considered near-full-length genome sequences sampled in the last 10 years, capturing contemporary circulating diversity (Fig. 6a and Extended Data Fig. 2). Within this framework, CHIKV diversity is subdivided into 21 categories. The three clades map to eight founding lineages (A–H). Specifically, the West African clade maps to A and B, ECSA maps to lineages D–H, and the Asian clade maps to lineage C (Fig. 6b). We define 12 sublineages across the founding lineages. The hierarchical system for CHIKV allows a maximum of three numeric sublevels before alias assignment. The current set of designated lineages does not exceed two sublevels (for example, lineages C.1.1 and E.6.1). Lineages were designated on a maximum likelihood phylogeny representing global CHIKV diversity since 2016, with designations largely informed by geographical clustering (Fig. 6c).

CHIKV sequences sampled before 2016 fall outside the nomenclature system and are designated O (Fig. 6b). Viral diversity within this category can be incorporated into the system as new founding lineages if re-observed in contemporary transmission, consistent with the criteria described in Fig. 3c. For example, a CHIKV genome sequence from a mosquito sampled in 2021 (accession number OQ054327) does not cluster with any of the eight founding lineages; however, if additional epidemiologically relevant genome data came to light, this diversity may be designated as an additional founding lineage.

## Discussion

Since the emergence of SARS-CoV-2, fine-scale nomenclature systems have been applied to other pathogens, and as genomic surveillance becomes increasingly integrated into outbreak response, this trend is likely to continue. Adhering to a shared set of core lineage-naming principles, such as those proposed here, ensures that lineage designations remain interpretable for public health scientists, without the overhead of learning or developing multiple parallel systems. It also allows the community to leverage the suite of computational tools already developed for SARS-CoV-2, saving time and investment. A lineage system for an emerging outbreak can capitalize on pre-existing software to explore new lineages, and on the Pango GitHub designation framework to define and share new lineages^7,20,34^.

A formalized Pango lineage nomenclature offers a flexible template for real-time genomic epidemiology across diverse viral families. Each pathogen presents unique biological and public health challenges; consequently, the principles laid out here are deliberately general and permit thresholds and criteria for lineage designation to be fine-tuned on a case-by-case basis. As we learn more about viral diversity, exceptions will inevitably arise, and nomenclature rules may need to remain as dynamic as the lineages they track. Nevertheless, the Pango lineage principles of utility, scalability, transparency and equity provide a strong foundation upon which to build future systems. As viral genomic data continue to inform outbreak control, coherent and accessible nomenclature will be vital for translating the complex genomic information into public health action.

## Methods

### Data acquisition and filtering

All CHIKV genome sequences were downloaded from NCBI on 30 March 2026 (*n* = 8,838). Thresholds of a minimum length of 10,000 nucleotides and a maximum N content of 0.2 were applied to the dataset, and records without sampling date information were excluded, leaving only near-full-length genomes. This resulted in a dataset of 3,175 genome sequences from 94 countries, covering a date range from 1952 to August 2025. The mean N content of the dataset was 0.0082. Sequence headers were harmonized using Raccoon (https://github.com/artic-network/raccoon).

### Phylogenetics and lineage designation

The sequences were aligned by mapping against the CHIKV RefSeq (accession ID NC_004162) using minimap2 v2.30-r1287 and converting the output to a multiple sequence alignment using gofasta v1.2.3 (refs. ^50,51^). A maximum likelihood phylogenetic tree was estimated using IQ-TREE v3.0.1 with an HKY substitution model, zero-length branches collapsed and 1,000 ultrafast bootstraps^52,53^. The tree was midpoint rooted, and clades were annotated in FigTree. To reflect more contemporary CHIKV diversity, the dataset was filtered to contain sequences sampled from 2016 onwards, leaving 2,299 sequences from 42 countries. A maximum likelihood phylogeny was estimated using IQ-TREE as described above^52,53^. Founding lineages were annotated on the phylogeny based on phylogenetic clustering, date and location information, and additional sublineages were defined where appropriate. Annotations were extracted using jclusterfunk v0.0.25 (https://github.com/rambaut/jclusterfunk). Detailed lineage descriptions and GenBank accession-to-lineage mappings are hosted on the CHIKV lineage GitHub repository at https://github.com/chikv-lineages/pango-designation.

### Reporting summary

Further information on research design is available in the Nature Portfolio Reporting Summary linked to this article.

## Supplementary information

Supplementary Information

Reporting Summary

Peer Review File

Supplementary Data 1 A maximum likelihood tree of high-quality CHIKV genome sequences on GenBank.

Supplementary Data 2 A maximum likelihood tree of high-quality CHIKV genome sequences on GenBank collected from 2016 onwards.

Supplementary Data 3 The complete set of sequence accessions used for the lineage designation and the corresponding lineage designation.

Supplementary Data 4 The data used to make the nomenclature Sankey diagrams, mapping the other nomenclatures to the Pango lineage system.

Supplementary Data 5 Detailed descriptions of each of the designated lineages, also hosted on GitHub.

## Data Availability

All data used in this paper were accessed from GenBank, and accessions of sequences used in the analysis are provided in the Supplementary Information and are also available at https://github.com/chikv-lineages/pango-designation. The CHIKV phylogenies, detailed lineage descriptions and GenBank accession numbers are also provided as accompanying source data for this paper.

## Extended data

**Extended Data Table 1:** Glossary of some commonly used terms to describe and characterise virus diversity below the species rank

Term	Description	Example
Serotype	A categorisation based on distinct antigenic or cross neutralisation properties.	Poliovirus^42^; Dengue Virus^43^; Foot-and-Mouth Disease Virus^44^
Genotype	A categorisation based on shared genetic characteristics, typically defined by a combination of specific alleles.	Hepatitis C Virus^45^; Hepatitis E virus^46^; Rotavirus A^3^; Noroviruse^47^; BK Polyomavirus^48^; Hendra virus^49^; Human adenovirus^50^; Measles virus^51^
Type	A genetically defined subdivision within a virus species. Type has a distinct use in taxonomy, referring to the exemplar used for definition of a taxon.	Rhinoviruses^42^; HPV^52^; Adenovirus^50^
Subtype	A genetically distinct subdivision of genotype or type, typically based on thresholds of genetic distance.	Hepatitis E virus^46^; HIV-1 subtypes (A-F)^2^; Dengue Virus^43^
Sub-subtype	A further subdivision within a subtype representing a distinct lineage that does not meet the criteria for designation as a subtype.	HIV-1^2^
Clade	A monophyletic group of viruses defined by a common ancestor on a phylogenetic tree.	Seasonal Influenza Viruses^53^; MPXV^8^; Nipah virus^54^; SARS-CoV-2^55^
Subclade	A monophyletic subdivision within a clade representing finer-scale evolutionary structure.	Seasonal Influenza viruses^53^; SARS-CoV-2^55^
Group	A category used variably to define groupings; may refer to major divisions or fine-scale divisions depending on context.	Rhinovirus^56^; HIV-1 groups (M, N & O)^2^; MPXV Clade Ia^57^; Rotavirus^3^
Genogroup	A categorisation based on phylogenetic clustering or genotype.	Norovirus^47^; Sapovirus^58^
Variant	An informal term describing a virus differing from a reference strain by one or more mutations. Certain terms have specific meanings (for example SARS-CoV-2 Variant of Concern).	Geminivirus^59^; SARS-CoV-2^19^; Ebolavirus^60^
Strain	A virus with demonstrable, heritable phenotypic differences, such as increased transmissibility or virulence.	Geminivirus^59^; Nipah virus^61^; West Nile virus^62^; Tick borne encephalitis virus^63^
Lineage	A group of viruses sharing a common ancestor, typically identified as a cluster within a phylogenetic tree. A lineage is also formally defined as part of the Pango lineage nomenclature system.	Dengue virus^64^; SARS-CoV-2^5^; Rabies virus^9^; West Nile virus^62^

**Extended Data Table 2:** The genotype annotations given to the 3,376 CHIKV genome sequences on Genbank

Genotype annotation	Count	Sample locations
Asia	9	Saint Martin; Trinidad and Tobago
Asian	317	Anguilla; Antigua and Barbuda; Argentina; Bahamas; Barbados; Brazil; British Virgin Islands; Cayman Islands; China; Dominica; French Guiana; Grenada; Guadeloupe; Guyana; Haiti; Indonesia; Jamaica; Kiribati; Mexico; Montserrat; Nicaragua; Paraguay; Philippines; Saint Kitts and Nevis; Saint Lucia; Saint Martin; Saint Vincent and the Grenadines; Samoa; Senegal; Singapore; Suriname; Thailand; Timor-Leste; Trinidad and Tobago; Turks and Caicos Islands; USA
Asiatico	15	Colombia
Brazilian	1	Argentina
ECS	1	India
ECSA	583	Australia; Bangladesh; Borneo; Brazil; Cambodia; China; Democratic Republic of the Congo; Djibouti; France; Gabon; Haiti; India; Italy; Kenya; Madagascar; Malaysia; Mayotte; Myanmar; Pakistan; Papua New Guinea; Republic of the Congo; Saudi Arabia; Singapore; South Korea; Thailand; USA
ECSA,	1	USA
ECSA-IOL	8	India; Malaysia
ECSA-iol	10	China
East-Central-South-Africa	41	Brazil
East-Central-South-African	907	Argentina; Brazil; Cameroon; India; Italy; Malaysia; Nicaragua; Paraguay; Senegal; Uruguay
East/Central/South	128	Brazil; Cote d’Ivoire; France; Malaysia; Paraguay
Eastern/Central/Southern	6	Pakistan
Indian	7	India;Sri Lanka
*Missing annotation*	1342	

There is a lack of standardisation across genotype usage, the geographic descriptors do not fully capture the current diversity and many of the sequence records lack a genotype annotation.

## Code availability

Custom code used to create the Sankey plots and ridge plot is available at https://github.com/chikv-lineages/pango-designation/tree/main/scripts.
