# Supplementary material for: A generalized lineage nomenclature for viral genomic epidemiology
Source: Nat Microbiol. 2026 Sep 30;11(10):2901–10. doi: 10.1038/s41564-026-02468-3 (PMC13716827; doi:10.1038/s41564-026-02468-3)
Supplement: Supplementary file 1 [file 41564_2026_2468_MOESM1_ESM.pdf]

nature microbiology

Supplementary information https://doi.org/10.1038/s41564-026-02468-3

A generalized lineage nomenclature for viral genomic epidemiology

In the format provided by the authors and unedited

Rules for the Designation and Naming of Pango Lineages (Template)

SECTION I. Criteria for designation of a new Pango lineage

1. A set of genome or sub-genomic sequences may be considered for the designation of a new lineage name if it exhibits the following essential characteristics:

   1a. At the time of designation, the set of sequences is expected to share a single common ancestor and represent a monophyletic or paraphyletic clade in a phylogeny representative of known diversity

   1b. The clade should be distinguished by at least X unambiguous evolutionary event (single nucleotide change, insertion/deletion, recombination or reassortment event).

   1c. The clade should contain a minimum of Y sequences.

   1d. The clade must include at least one internal node and therefore cannot be solely composed of a single polytomy. In the absence of genetic resolution, in some cases a time tree may be used to resolve internal nodes. Thus, a lineage is expected to be consistent with a signal of onward transmission.

2. A new non-recombinant lineage designation is expected to also represent one or more events of epidemiological significance

   2a. A non-exhaustive list of possible events are as follows:

      (i) The clade may represent inferred movement of the virus into a new country or region (founder event).

      (ii) The clade may be observed to be growing rapidly and/or strongly increasing in frequency compared to other co-circulating lineages.

      (iii) The clade may be associated with observed or predicted changes in phenotypes including, but not limited to, transmissibility, immunogenicity, or pathogenicity.

      (iv) The clade may carry a set of multiple mutations of particular biological interest or concern. Note that, in most cases, the presence of a single specific mutation, by itself, will not be considered sufficient to warrant a new lineage designation.

      (v) A circulating clade initiated by a recombination or reassortment event.

   2b. If a clade becomes the subject of exceptional interest, and a new lineage designation would greatly clarify reference to and discussion of that clade, then a new designation may be considered even if none of the event conditions in 2.2a are met. The conditions in section I.1 should still be met.

3. The essential characteristics in section 1.1 are *necessary but not sufficient* for the

designation of a new lineage. Therefore, not all phylogenetic clades that meet the conditions in section 1.1 will be given a Pango lineage designation.

3a. Lineage designation is at the discretion of the Pango designation committee (See governance section V) and its interpretation of clade characteristics. The number of available genomes across locations and times may be taken into account when interpreting these designation rules.

3b. In most instances it is expected that the number of high quality genomes required to designate a lineage will be greater than the minimum number defined in I.1c.

SECTION II. Definition of a hierarchical alpha-numeric system for lineage names

Each Pango lineage designated according to the guidelines in Section 1 is given its own unique lineage name. Pango lineage names are based on phylogenetic structure, but are intended to convey only partial, local information about ancestor-descendant relationships.

1. Syntax

1a. Lineage names are constructed from an alphabetical prefix and a numerical suffix. Although lineage names are case-insensitive, the alphabetical prefix is typically capitalised.

1b. Any letter in the Latin alphabet[1], may be used (singly or in combination) within the prefix of standard lineages, except for I, O and X. The letters I and O are omitted to avoid confusion with the numerals 1 and 0. X is reserved for the names of recombinant lineages (see II.1j).

1c. Each full stop (or period or dot) within the numerical suffix represents "descendant of" and is applied when one ancestor of the lineage can be clearly identified (see II.1g).

Example: Lineage B.1.1.7 is the seventh named descendant of lineage B.1.1, which in turn was the first named descendant of lineage B.1.

1d. In order to avoid excessively long lineage labels, the numerical suffix has a maximum of Z hierarchical levels (primary, secondary and tertiary suffixes). Descendants of lineages with tertiary suffixes are assigned to the next available permitted alphabetical prefix, in alphabetical order. This new prefix acts as an *alias* for the name of the parental lineage.

Example: The first named descendant of lineage B.1.1.1 is not named B.1.1.1.1 but is instead named C.1. The prefix C therefore serves as an alias for B.1.1.1.

1e. Details of each *alias* must be provided in the current lineage description list, so that the ancestral relationships of all Pango lineages can be reconstructed unambiguously.[2]

1f. Most lineages are *standard lineages*. The names of *standard lineages* must contain both an alphabetical prefix and a numerical suffix. This captures the fact that the prefix of each standard lineage represents a single unambiguous ancestor.

1g. Some lineages do not have a single unambiguous parental lineage and are called *special case lineages*. At present there are two types of special case lineages. The first refers to founding lineages, which describe the relevant clusters present at the initial time point of a Pango nomenclature. The second type comprises recombinant lineages that, by definition, have more than one parental lineage (see II.1j). A special case lineage name can be used without a numerical suffix. Descendent lineages of special case lineages acquire numerical suffixes according to the procedure set out in II.1c-II.1e.

1h. After all available single character prefixes are exhausted, designations continue with two-character prefixes, beginning with the next available letter, and so on.

Example: Prefix AA is the next available prefix after Z, and BA is the next available prefix after AZ, and AAA is the next available prefix after ZZ.

1i. The next available prefix is determined by reference to the current lineage description list. The list must be updated promptly following a new designation.[3]

1j. Recombinant lineages are given alphabetical prefixes beginning with the letter X. With the addition of this condition, recombinant lineages are given the next available prefix according to II.1h. Incrementation of the prefix for new recombinants is independent of that for new non-recombinants, and determined by reference to the current lineage description list (see II.1i).

Example: The first detected recombinant lineage is named XA, followed by XB, XC,..., XAA, XAB, etc.

1k. Recombinant lineages are *special case lineages* and used without a numerical suffix (see II.1g). Non-recombinant descendent lineages of recombinant lineages acquire numerical suffixes, according to II.1c-II.1e.

Example: The first non-recombinant descendant lineage of XA is named XA.1 because it has one unambiguous parental lineage. The usual rules of aliasing also apply, hence XA.1.1.1.1 becomes AJ.1, if AJ is the next available top-level prefix.

1l. As recombinant lineage names lack information about their putative parental lineages, this information (which may be uncertain) is added to the lineage description list.

SECTION III. Implementation and revision

1a. Sequences assigned to Pango lineages are aligned relative to the reference sequence, which by convention is that of Wuhan-Hu-1 (GenBank: MN908947.3), and genetic changes are called relative to that reference. Consequently, positional homology is implied.

1b. Lineages are designated only if they descend from the common ancestor of the SARS-CoV-2 pandemic in humans. Pango does not name or classify pre-pandemic viruses recovered from other species. However, if a human pandemic lineage moves into another host species (or thereafter back into humans) then Pango lineages may be designated for each cross-species transmission, in accordance with I.2a.

1c. Retrospective renaming of lineages should be avoided wherever possible.

1d. The Pango nomenclature is dynamic and intended to capture active, ongoing transmission. Therefore each lineage has a status, which is recorded in the lineage description list. This status must be one of the categories listed below and represents the current observation state of the lineage:

ACTIVE: last sampled within last 3 months

UNOBSERVED: last sampled seen 3-9 months ago

INACTIVE: not sampled for 9 months

WITHDRAWN: name not in use

UNDEFINED: see III.1g

1e. A lineage may be withdrawn for reasons of sample quality, incorrect metadata, clarification, phylogenetic construction errors, or other matters arising.

1f. Once withdrawn, a lineage name cannot be reused and reassigned, and should be permanently marked as having WITHDRAWN status in the lineage description list.

1g. If a designated lineage needs to be split into multiple lineages (e.g. through phylogenetic revision following additional sampling) then its descendent lineages are given the next available lineage suffixes of their parent, or the next available aliases, as appropriate following the procedure in Section II. A note of the change must be added to the lineage description list.

1h. Revisions (for whatever reason) may lead to the number of sequences in a designated lineage falling below the number required to designate a new lineage (see I.1.1c). The names of such lineages are retained, and they remain in the lineage description list, regardless of the number of sequences that they contain. If the remaining number of sequences is zero, then the lineage name remains and its lineage status is UNDEFINED, not

withdrawn.

1i. In the event that a lineage is erroneously designated with a label that is already in use, the more recently designated lineage is re-designated using the next available name according to Section II, and a note is added to the lineage description list.

1j. Should a valid recombination event be overlooked or an invalid recombinant be designated, in neither case should the lineage name be changed, but a note must be added to the lineage description list upon recognition of the misdesignation.

SECTION IV. Designated sequences

1a. A sequence designation list comprises a list of genomes sequences that have been designated to a Pango lineage.

1b. Each designated genome sequence belongs to one, and only one, Pango lineage.

1c. Sequences must be of high coverage to be included in the sequence designation list. High High coverage means that <X% of nucleotides sites in the whole genome (excluding UTRs) are represented by IUPAC ambiguity codes.

1.d Not all sequences that meet the condition in IV.1c will be necessarily included in the sequence designation list. For example, not all members of lineages that contain large numbers of SARS-CoV-2 genomes will be included.

1.e If a genome sequence that is not in the sequence designation list has exactly the same SARS-CoV-2 nucleotide sequence as a member of the sequence designation list, then it can be safely assumed that the lineage of the former is equal to that of the latter.

SECTION V. Governance

Any Pango lineage nomenclature should have a system in place for maintenance beyond the initial diversity characterisation, with a transparent governance committee that includes representation from the stakeholders who most benefit from the existence of the nomenclature.

---

[1] The ISO basic Latin alphabet (ISO/IEC 646)

[2] Currently available at https://github.com/cov-lineages/pango-designation/blob/master/full_alias_key.txt

[3] Currently available at https://github.com/cov-lineages/pango-designation/blob/master/full_alias_key.txt
