# Supplementary material for: A generalized lineage nomenclature for viral genomic epidemiology
Source: Nat Microbiol. 2026 Sep 30;11(10):2901–10. doi: 10.1038/s41564-026-02468-3 (PMC13716827; doi:10.1038/s41564-026-02468-3)
Supplement: Supplementary file 3 [file 41564_2026_2468_MOESM3_ESM.pdf]

nature portfolio

Peer Review File

A generalised lineage nomenclature for viral genomic epidemiology

Corresponding Author: Dr Áine O'Toole

Version 0:

Decision Letter:

3rd March 2026

Dear Dr O'Toole,

Thank you for your patience while your Perspective entitled "A generalised lineage nomenclature for viral genomic epidemiology" has now been seen by 3 referees. As you will see from their comments at the end of this email, while they find your article of interest, they have raised points that need to be addressed before we can make a decision on publication. We will need to consider your response to these concerns in the form of a revised manuscript, accompanied by a separate list to explain your revisions.

In light of the comments raised by our referees (see below), we have decided that we would like to consider your article as Perspective. This format will allow for the additional discussions and explanations requested by our reviewers - beyond the field of virology - particularly mentioned by referee #3.

Perspectives are about 4,000 words long (excluding the references, text boxes and figure legends), can have up to 100 references and up to 7 display items (including figures, boxes and tables).

A separate point-by-point response to ALL of the concerns raised by the referees should be included with the revised manuscript, in each case describing what changes have been made to the manuscript or, alternatively, if no action has been taken, providing a compelling argument for why that is the case.

Please add a Acknowledgements and Conflict of interest section, if you haven not yet.

Nature Portfolio defines Competing Interest (CI) as financial and non-financial interests (including but not limited to funding, employment, stocks, shares, patents, personal or professional relationships with individuals or institutions, and unpaid membership advocacy) that could be perceived to directly undermine the objectivity, integrity, and value of a publication, or could be seen as having an influence on the judgments and actions of authors with regard to objective data presentation, analysis, and interpretation.

We very much hope that you will be able to address these comments of our referees and would like to invite you to revise your manuscript accordingly.

When you are ready, please use the link below to submit the revised version (with the changes clearly marked).

Link Redacted revising your paper:

- include a point-by-point response to our referees. Please include your response to the editorial suggestions in your cover letter, and please upload your response to the referees as a separate document.

- state in a cover note the length of the text, methods and legends; the number of references and the number of display items (figures, tables and uncaptioned molecular structures).

Please ensure that all correspondence is marked with your Nature Microbiology reference number in the subject line.

Nature Microbiology is committed to improving transparency in authorship. As part of our efforts in this direction, we are now requesting that all authors identified as 'corresponding author' on published papers create and link their Open Researcher and Contributor Identifier (ORCID) with their account on the Manuscript Tracking System (MTS), prior to acceptance. This applies to primary research papers only. ORCID helps the scientific community achieve unambiguous attribution of all scholarly contributions. You can create and link your ORCID from the home page of the MTS by clicking on 'Modify my Springer Nature

account'. For more information please visit please visit <a
href="http://www.springernature.com/orcid">www.springernature.com/orcid.

Please note, Nature Microbiology publishes research articles and non-research articles such as Reviews, News & Views and Comments. Non-research articles published in Nature Microbiology can only be published using the subscription publication route; we do not offer an immediate gold open access (OA) publication option for non-research content. In contrast to research articles, non-research articles do not include original (previously unpublished) research findings and may only contain minimal re-analyses of published data. The vast majority of funders with OA mandates only require research that they have funded to be published OA, meaning that most authors submitting Reviews, Perspectives, Comments, News &Views, Correspondence to Nature Microbiology, do not need to take any actions to make those types of content openly accessible. The UKRI, for example, has confirmed that narrative reviews are out of the scope of their policy.

I would appreciate it if you could tell me if you think you will be able to submit a revised manuscript, and also the likely timescale.

I look forward to hearing from you soon.

Kind regards,

[redacted]

****
Reviewer Expertise:

Referee #1: Taxonomy, virology
Referee #2: Evolution, ecology, virology
Referee #3: Vaccines, emerging viruses

Reviewer Comments:

Reviewer #1 (Remarks to the Author):

I enjoyed reading this. The authors very clearly describe pros and cons of the Pango system and make a very good case for its continued use and possible expansion. The paper is written very nicely and the figures clarify all the discussed concepts; in particular I appreciated the very clear statements on when this system would not be useful and the role of the various communities in further development.
Suggestions for improvement:

- Title etc.: maybe add epizootics/epizootiology? You write later that it would work in that context at all
- Abstract, "recent epidemics being accompanied by large numbers of viral genetic sequences" is a bit awkward. First because the sentence starts with "pathogen" and there are other pathogens than viruses and second because of course any viral outbreak is associated with viruses and therefore viral "sequences" (nucleic acids). Maybe rewrite this according to the lines of recent epidemics being better characterizable due to increased sequencing and therefore increases availability of information?
- Abstract, "Effective international communication of viral diversity requires a scalable, adaptable nomenclature" … for what? For all those sequences (previous sentence)? I don't think that is what you mean, given that the paper is about lineages?
- Main, "The International Committee on Taxonomy of Viruses (ICTV) classifies and names viruses down to the species level". This is incorrect. The ICTV does *not* name viruses – the community does. The ICTV only names taxa, down to the species rank (not level). Please clarify this according to the lines of "The ICTV classifies viruses into taxa … and is responsible for taxon nomenclature … The community names viruses and assigns virus name abbreviations, often in consultation with ICTV Study Groups … Description [*not* "classification] of the diversity of viruses (e.g., strains, variants, isolates, genotypes, serotypes…) has been historically undertaken by…". Please abstain from using the term "subspecies" anywhere as this term implies a rank that does not exist in virus taxonomy (as opposed to zoology/botany). If you must, use "sub-species", but it be best to not go in that direction at all (African Americans are not "subspecies" or "sub-species" of humans…!).
- Main, "including type, subtype, genotype, group, strain and clade". This is an opportunity to point out how confusing these terms are. Type, for instance, has a specific meaning in most taxonomies, referring to the particular exemplar used for a taxon definition (in zoology/botany/bacteriology). Strain, in virology, is actually defined as a *phenotypic*, i.e., not genetic, grouping. For instance, a tick-borne encephalitis virus isolate that does not cause encephalitis (99.99% of TBEVs do) but instead causes hemorrhagic fever has been described and the change in pathogenesis was tracked down to two nt changes over the entire genome. In a phylogenetic tree, this virus is not special and would simply be an isolate in a clade. But it is a different strain than all the other viruses in that clade. If too complicated and confusing, maybe just delete "strain"?
- Main, "including for mpox virus" – the virus continues to be called "monkeypox virus (MPXV)". Only the disease has been renamed (and only within the WHO framework)
- Main, "respiratory syncytial virus (RSV)". Please change to the current name "human respiratory syncytial virus (HRSV)"
- Principles, "different modes of classification" – it would be better to replace classification with another term. Only the ICTV classifies in the strict sense.
- Principles, "full genomes". Please change to "coding-complete/complete" genomes. By various standards published in recent years, "full" is reserved for subpopulation characterization of a virus in culture
- Principles, "or partial subgenomic". Maybe delete "subgenomic"? To me" subgenomic" implies mRNAs etc. derived from genomes during viral replication

- Principles, “may be lower in slower-evolving species” – please replaced species with viruses. Viruses do evolve; species definitions are made and changes by humans
- Definition, “For reassorting viruses, in which virus genome segments follow distinct evolutionary ancestries, a Pango lineage nomenclature can be anchored to a single representative segment, the one most relevant for transmission or antigenicity”: please clarify that then you may leave overarching evolutionary connections? For many RNA viruses, the glycoproteins are proteins most relevant for transmission and antigenicity – however, these proteins mostly do not interact with any other viral proteins (i.e., do not need to coevolve) and hence their ORFs are the easiest ones to be swapped (which is also why pseudotype systems work so nicely). To me this means that glycoprotein genes are the least informative about viral macroevolution as opposed to, for instance, the RdRP (and via their interaction requirements, typically the entire replication module) and therefore, for depicting virus evolution, I would focus on segments encoding RdRP and ignore GP.
- Definition, “however some DNA viruses” – should this be more specifically dsDNA viruses? ssDNA viruses evolve quite rapidly
- Definition, “from the _Mastomys_ reservoir” – please change to “from the mastomys reservoir” (lower case, no italics). “Mastomys” are specific rodents, such as “rats”. _Mastomys_ is a genus to which mastomys are assigned, just like rats are assigned to genus _Rattus_
- Definition, “track viral diversity in species other than”. Please change to “animals other than” (or mammals, vertebrates etc.)
- Lessons, “The SARS-CoV-2 pandemic” – please change to “The COVID-19 pandemic”
- I really like Suppl Fig 1 – could this be moved into the main text?

Reviewer #2 (Remarks to the Author):

This manuscript presents a generalised framework for implementing a Pango-style, phylogeny-based lineage nomenclature for viral genomic surveillance. The authors discuss applicability across outbreak types and viral taxa, and reflect on lessons learned from six years of implementation during the COVID-19 pandemic. The paper is timely, conceptually important, and likely to serve as a reference point for future outbreak genomics efforts. The discussion of equity and sampling bias reflects real-world challenges and strengthens the manuscript. I only have some minor suggestions which I hope will improve the manuscript:
- More explicit comparison with existing systems would be useful, for example with some long-standing clade systems (e.g. influenza virus, HIV-1 etc.), which are only briefly mentioned. Perhaps a table comparing the granularity and how dynamic they are compared to Pango.
- The manuscript raises the sustainability issue that “no structure or organisation exists to fund their establishment and maintenance”. This is a critical point. Given that lineage systems can influence public health policy, the governance model could be further elaborated perhaps with some suggestions of how a funded model could work.
- Occasionally “Pango system,” “Pango nomenclature,” and “Pango lineage system” are used interchangeably. Consider standardising usage.
- Some hyphenation inconsistencies (e.g. sustained-human vs sustained human).
- I suggest expanding the text on the ICTV/virus naming. For example, Pango may be able to influence discussions within ICTV about how fine-scale viral diversity should be named and communicated. This seems like it would be helpful for the virology community compared to the currant system.

Reviewer #3 (Remarks to the Author):

Colquhoun and colleagues highlight the emergence of the Pango lineage nomenclature system and its applicability, generalizability, and implementation for future genomic surveillance for additional viruses. The article is informative but is some places it is written in a field specific manner which can be hard for the interdisciplinary readership of Nat Micro to comprehend. Some suggestions for the authors follow.

1. A table or figure highlighting other methods that have been used for virus classification and how they compare to Pango will be useful for the readership.

2. Can Pango-style nomenclature framework be expanded beyond viruses to bacteria or cancer mutations or mutations associated with genetic disorders and autoimmune diseases? Why yes or no?

3. How will Pango framework address human-animal and animal-human transmission? Authors mention growth advantage, recombination, immune escape and vaccine evasion among others for SARS-CoV-2, but highlighting the ability to monitor zoonotic and zooanthroponotic transmission and adaptation will strengthen the approach (if the virus evolves between different hosts). SARS-CoV-2 infected several mammalian species, and the evolutionary source or mechanism for various VOCs remain unknown.

4. If lineages were to be assigned an ‘event’ like epidemiological event, how does one confidently rule out the possibility of other overlapping but unreported parameters, like evolution in a new host in this new region or region? How does one ensure the accuracy of assigning an event to a node?

5. Is Pango truly only useful in the event of large outbreaks? Can it improve upon others ways outbreaks are monitored and can it be used to connect different outbreaks over time for the same pathogen? The authors touch upon this but the limitations of why Pango cannot be used or is not particularly useful for slow evolving viruses or for smaller, disconnected outbreaks can be better explained.

****

Version 1:

Decision Letter:

Our ref: NMICROBIOL-26010309A

3rd June 2026

Dear Áine,

Thank you for submitting your revised manuscript "A generalised lineage nomenclature for viral genomic epidemiology" (NMICROBIOL-26010309A). It has now been seen by the original referees and their comments are below. The reviewers find that the paper has improved in revision, and therefore we'll be happy in principle to publish it in Nature Microbiology, pending minor revisions to satisfy the referees' final requests and to comply with our editorial and formatting guidelines.

If the current version of your manuscript is in a PDF format, please email us a copy of the file in an editable format (Microsoft Word or LaTex)-- we can not proceed with PDFs at this stage.

We are now performing detailed checks on your paper and will send you a checklist detailing our editorial and formatting requirements in about a week. Please do not upload the final materials and make any revisions until you receive this additional information from us.

Thank you again for your interest in Nature Microbiology Please do not hesitate to contact me if you have any questions.

Sincerely,

[redacted]

Reviewer #1 (Remarks to the Author):

All my minor concerns have been addressed satisfactorily. Nice work.

Reviewer #2 (Remarks to the Author):

The authors have addressed all of my previous comments and I am satisfied with this version of the manuscript.

Reviewer #3 (Remarks to the Author):

The authors have addressed all my comments by performing additional analysis, along with edits within the text.

Version 2:

Decision Letter:

28th July 2026

Dear Áine,

I am pleased to accept your Analysis "A generalised lineage nomenclature for viral genomic epidemiology" for publication in Nature Microbiology. Thank you for having chosen to submit your work to us and many congratulations.

Over the next few weeks, your paper will be copyedited to ensure that it conforms to Nature Microbiology style. We look particularly carefully at the titles of all papers to ensure that they are relatively brief and understandable.

Once your paper is typeset, you will receive an email with a link to choose the appropriate publishing options for your paper and our Author Services team will be in touch regarding any additional information that may be required. Once your paper has been

scheduled for online publication, the Nature press office will be in touch to confirm the details.

You may wish to make your media relations office aware of your accepted publication, in case they consider it appropriate to organize some internal or external publicity. Once your paper has been scheduled you will receive an email confirming the publication details. This is normally 3-4 working days in advance of publication. If you need additional notice of the date and time of publication, please let the production team know when you receive the proof of your article to ensure there is sufficient time to coordinate. Further information on our embargo policies can be found here:
https://www.nature.com/authors/policies/embargo.html

After the grant of rights is completed, you will receive a link to your electronic proof via email with a request to make any corrections within 48 hours. If, when you receive your proof, you cannot meet this deadline, please inform us at rjsproduction@springernature.com immediately. You will not receive your proofs until the publishing agreement has been received through our system

Due to the importance of these deadlines, we ask you please us know now whether you will be difficult to contact over the next month. If this is the case, we ask you provide us with the contact information (email, phone and fax) of someone who will be able to check the proofs on your behalf, and who will be available to address any last-minute problems.

Acceptance of your manuscript is conditional on all authors' agreement with our publication policies (see https://www.nature.com/nmicrobiol/editorial-policies). In particular your manuscript must not be published elsewhere.

Authors may need to take specific actions to achieve compliance with funder and institutional open access mandates. If your research is supported by a funder that requires immediate open access (e.g. according to Plan S principles or the NIH public access policy) then you should select the gold OA route, and we will direct you to the compliant route where possible. Because authors warrant under our subscription licensing terms that they haven't committed to licensing any version of their article under a licence inconsistent with the terms of our agreement – including the applicable embargo period – publication under the subscription model isn't suitable for authors whose funders require no embargo.

If you have any questions about our publishing options, costs, Open Access requirements, or our legal forms, please contact ASJournals@springernature.com

An online order form for reprints of your paper is available at https://www.nature.com/reprints/author-reprints.html. All co-authors, authors' institutions and authors' funding agencies can order reprints using the form appropriate to their geographical region.

We welcome the submission of potential cover material (including a short caption of around 40 words) related to your manuscript; suggestions should be sent to Nature Microbiology as electronic files (the image should be 300 dpi at 210 x 297 mm in either TIFF or JPEG format). Please note that such pictures should be selected more for their aesthetic appeal than for their scientific content, and that colour images work better than black and white or grayscale images. Please do not try to design a cover with the Nature Microbiology logo etc., and please do not submit composites of images related to your work. I am sure you will understand that we cannot make any promise as to whether any of your suggestions might be selected for the cover of the journal.

You can now use a single sign-on for all your accounts, view the status of all your manuscript submissions and reviews, access usage statistics for your published articles and download a record of your refereeing activity for the Nature journals.

To assist our authors in disseminating their research to the broader community, our SharedIt initiative provides you with a unique shareable link that will allow anyone (with or without a subscription) to read the published article. Recipients of the link with a subscription will also be able to download and print the PDF.

As soon as your article is published, you will receive an automated email with your shareable link.

Congrats again to you and your co-authors! I am looking forward to seeing your paper published.

With kind regards,

[redacted]

P.S. Click on the following link if you would like to recommend Nature Microbiology to your librarian
http://www.nature.com/subscriptions/recommend.html#forms

** Visit the Springer Nature Editorial and Publishing website at www.springernature.com/editorial-and-publishing-jobs for more information about our career opportunities. If you have any questions please click here.**

Open Access This Peer Review File is licensed under a Creative Commons Attribution 4.0 International License, which permits use, sharing, adaptation, distribution and reproduction in any medium or format, as long as you give appropriate credit to the original author(s) and the source, provide a link to the Creative Commons license, and indicate if changes were made.
In cases where reviewers are anonymous, credit should be given to 'Anonymous Referee' and the source.
The images or other third party material in this Peer Review File are included in the article's Creative Commons license, unless indicated otherwise in a credit line to the material. If material is not included in the article's Creative Commons license and your intended use is not permitted by statutory regulation or exceeds the permitted use, you will need to obtain permission directly from the copyright holder.
To view a copy of this license, visit https://creativecommons.org/licenses/by/4.0/

NMICROBIOL-26010309: Response to reviewers

Key:

- *Reviewer's comments in italics and grey*
- Author's response in black

Editor's comments

- include a point-by-point response to our referees. Please include your response to the editorial suggestions in your cover letter, and please upload your response to the referees as a separate document.

- state in a cover note the length of the text, methods and legends; the number of references and the number of display items (figures, tables and uncaptioned molecular structures).

Reviewer #1:
Expertise: Taxonomy, virology

I enjoyed reading this. The authors very clearly describe pros and cons of the Pango system and make a very good case for its continued use and possible expansion. The paper is written very nicely and the figures clarify all the discussed concepts; in particular I appreciated the very clear statements on when this system would not be useful and the role of the various communities in further development.

We appreciate the positive feedback and for taking the time to review our manuscript. We also appreciate the attention to detail, which has helped us improve the clarity and precision of the terminology used throughout the manuscript.

Suggestions for improvement:

- Title etc.: maybe add epizootics/epizootiology? You write later that it would work in that context at all

This is a good point, and we realise that we have framed this manuscript very much with human outbreaks in mind. Partly, this is because human outbreaks are far more likely to be sampled and sequenced at the scale and rate that would be amenable to a fine-scale lineage system like Pango. This may change in the future and we are seeing a greater trend towards genomics use in epizootics (e.g. the avian flu outbreak in cattle in the USA). Saying that, we feel this framework will be most useful for human viruses for now, so will keep the title as is, although we have added reference in the text to Pango's application to epizootics as well.

- Abstract, "recent epidemics being accompanied by large numbers of viral genetic sequences" is a bit awkward. First because the sentence starts with "pathogen" and there are other pathogens than viruses and second because of course any viral outbreak is associated with viruses and therefore viral "sequences" (nucleic acids). Maybe rewrite this according to the lines of recent epidemics being better characterizable due to increased sequencing and therefore increases availability of information?

We agree, and have rephrased the sentence to now be more specific and read: "Pathogen genomics has become increasingly integrated into outbreak response, with recent virus epidemics better characterised by large numbers of viral genetic sequences."

- Abstract, "Effective international communication of viral diversity requires a scalable, adaptable nomenclature" … for what? For all those sequences (previous sentence)? I don't think that is what you mean, given that the paper is about lineages?

We have clarified in the text that we are specifically talking about viral genetic diversity here.

*- Main, "The International Committee on Taxonomy of Viruses (ICTV) classifies and names viruses down to the species level". This is incorrect. The ICTV does *not* name viruses – the community does. The ICTV only names taxa, down to the species rank (not level). Please clarify this according to the lines of "The ICTV classifies viruses into taxa … and is responsible for taxon nomenclature … The community names viruses and assigns virus name abbreviations, often in consultation with ICTV Study Groups …*

We have amended the text to now read "The International Committee on Taxonomy of Viruses (ICTV) classifies viruses down to the species rank".

*Description [*not* "classification] of the diversity of viruses (e.g., strains, variants, isolates, genotypes, serotypes…) has been historically undertaken by…". Please abstain from using the term "subspecies" anywhere as this term implies a rank that does not exist in virus taxonomy (as opposed to zoology/botany). If you must, use "sub-species", but it be best to not go in that direction at all (African Americans are not "subspecies" or "sub-species" of humans…!).*

We have now amended the text to read "Description of viral diversity below the species rank has historically been undertaken by the scientific community, and virus variation has been subdivided…"

*- Main, "including type, subtype, genotype, group, strain and clade". This is an opportunity to point out how confusing these terms are. Type, for instance, has a specific meaning in most taxonomies, referring to the particular exemplar used for a taxon definition (in zoology/botany/bacteriology). Strain, in virology, is actually defined as a *phenotypic*, i.e., not genetic, grouping. For instance, a tick-borne encephalitis virus isolate that does not cause encephalitis (99.99% of TBEVs do) but instead causes hemorrhagic fever has been*

described and the change in pathogenesis was tracked down to two nt changes over the entire genome. In a phylogenetic tree, this virus is not special and would simply be an isolate in a clade. But it is a different strain than all the other viruses in that clade. If too complicated and confusing, maybe just delete "strain"?
We agree this is an opportunity to showcase the confusing ecosystem of terms used! We have removed "strain" for simplicity in this introductory section text, however have added a supplementary table (Supplementary table 1) that describes in more detail some of the terms commonly used to characterise virus diversity below the species rank and gives examples of where the terms have been applied.

- Main, "including for mpox virus" – the virus continues to be called "monkeypox virus (MPXV)". Only the disease has been renamed (and only within the WHO framework)

As the reviewer says earlier, it is the community that names viruses and we believe the community is increasingly preferring mpox virus (e.g. Lusamaki et al 2025 Cell, Yu et al 2023 Viruses). We believe the reasons that prompted WHO to change the name of the disease are correct and also apply to the name of the virus.

- Main, "respiratory syncytial virus (RSV)". Please change to the current name "human respiratory syncytial virus (HRSV)"
Complete.

- Principles, "different modes of classification" – it would be better to replace classification with another term. Only the ICTV classifies in the strict sense.
Have changed the sentence to now read "different modes of categorisation"

- Principles, "full genomes". Please change to "coding-complete/complete" genomes. By various standards published in recent years, "full" is reserved for subpopulation characterization of a virus in culture
The text now reads: "The data in question can represent either complete genomes …"

- Principles, "or partial subgenomic". Maybe delete "subgenomic"? To me" subgenomic" implies mRNAs etc. derived from genomes during viral replication
We have made the following change: "The data in question can represent either complete genomes or partial genome sequences, however, greater granularity will be achieved if complete genomes are used"

- Principles, "may be lower in slower-evolving species" – please replaced species with viruses. Viruses do evolve; species definitions are made and changes by humans
We have changed the text to "viruses".

- Definition, "For reassorting viruses, in which virus genome segments follow distinct evolutionary ancestries, a Pango lineage nomenclature can be anchored to a single representative segment, the one most relevant for transmission or antigenicity": please clarify that then you may leave overarching evolutionary connections? For many RNA viruses, the glycoproteins are proteins most relevant for transmission and antigenicity – however, these proteins mostly do not interact with any other viral proteins (i.e., do not need to coevolve) and hence their ORFs are the easiest ones to be swapped (which is also why pseudotype systems work so nicely). To me this means that glycoprotein genes are the least informative about viral macroevolution as opposed to, for instance, the RdRP (and via their interaction requirements, typically the entire replication module) and therefore, for depicting virus evolution, I would focus on segments encoding RdRP and ignore GP.

In our generalisation of Pango lineages, the priority remains applicability to public health and epidemiology. We are not trying to depict virus macro evolution, but are proposing Pango lineages as a framework to track virus transmission. In this case, tracking antigenicity and immune evasion is the priority, however we recognise that the system is flexible and that priorities may vary by community. If a specific virus community would rather track based on RdRP, this can also be achieved with Pango.

We have modified the text to clarify that our recommendation to select the most antigenically relevant segments is in the case of genomic epidemiology (this is also how the flu community tracks segments). Further, the external proteins often evolve fastest, and thus provide most resolution for fine-scale tracking over the course of an outbreak.

The text now reads: "For reassorting viruses, in which virus genome segments follow distinct evolutionary ancestries, a Pango lineage nomenclature can be anchored to a single representative segment. The choice of segment can be adapted for the intended application; for example an appropriate choice for genomic epidemiology will be the one most relevant for transmission or antigenicity, which is often the fastest evolving so can provide the most resolution over short timescales."

- Definition, "however some DNA viruses" – should this be more specifically dsDNA viruses? ssDNA viruses evolve quite rapidly

You are correct that we are mostly talking about dsDNA viruses in this case and have added this to the text. It now reads "however some DNA viruses, such as double stranded DNA viruses, evolve too slowly to be informative".

*- Definition, "from the _Mastomys_ reservoir" – please change to "from the mastomys reservoir" (lower case, no italics). "Mastomys" are specific rodents, such as "rats". _Mastomys_ is a genus to which mastomys are assigned, just like rats are assigned to genus _Rattus_*

We have made this change.

- Definition, "track viral diversity in species other than". Please change to "animals other than" (or mammals, vertebrates etc.)
We have amended the text.

- Lessons, "The SARS-CoV-2 pandemic" – please change to "The COVID-19 pandemic"
We have amended the text.

- I really like Suppl Fig 1 – could this be moved into the main text?
We have moved this figure to the main text, it is now Figure 5. We have also now included a worked version of a Pango nomenclature system for yellow fever virus, highlighting the decision process detailed in this figure (Figure 6).

Reviewer #2:
Expertise: Evolution, ecology, virology

This manuscript presents a generalised framework for implementing a Pango-style, phylogeny-based lineage nomenclature for viral genomic surveillance. The authors discuss applicability across outbreak types and viral taxa, and reflect on lessons learned from six years of implementation during the COVID-19 pandemic. The paper is timely, conceptually important, and likely to serve as a reference point for future outbreak genomics efforts. The discussion of equity and sampling bias reflects real-world challenges and strengthens the manuscript. I only have some minor suggestions which I hope will improve the manuscript:
We thank the reviewer for their thoughtful comments and their time spent reviewing the manuscript.

- More explicit comparison with existing systems would be useful, for example with some long-standing clade systems (e.g. influenza virus, HIV-1 etc.), which are only briefly mentioned. Perhaps a table comparing the granularity and how dynamic they are compared to Pango.

We have created a table (Supplementary Table 1) that describes some of the commonly used systems and nomenclature terms that characterise virus diversity below the species rank. Where appropriate, we have included reference to the relative granularity of these systems (e.g. clade & sub-clade, type & sub-type), however from our review of the various terms it appears that a specific granularity for a given term is can be ill-defined across viruses (e.g. group is used as both a broad scale and fine scale descriptor).

Similarly, the Pango lineage nomenclature has a dynamic granularity that can be modified to fit particular purposes. For instance, one could enact a Pango system for HIV-1 with the same granularity as the current system, with a coarser granularity, or with a finer-scale granularity. In practice, Pango lineages will often be finer-scale than clades (as shown in Figure 4), but this is dependent on community need, sampling density and availability of data. We have provided this table however have avoided artificially placing a bound on the flexibility of the Pango framework.

We have also now given a specific example of existing nomenclature for chikungunya virus, and how Pango could be applied to help improve consistency of naming. In Figure 6 and Supplementary Figure 2 we explicitly compare existing clade/genotype/subgenotype names and how they could be converted to a Pango lineage system.

- The manuscript raises the sustainability issue that "no structure or organisation exists to fund their establishment and maintenance". This is a critical point. Given that lineage systems can influence public health policy, the governance model could be further elaborated perhaps with some suggestions of how a funded model could work.

We thank the reviewer for raising this important point. We agree that sustainability and governance are critical considerations for lineage nomenclature systems. In developing the SARS-CoV-2 Pango lineage committees, we wanted to ensure broad international representation and to include contributors of genomic data in decision making processes. From our experience, the expertise for maintaining such a model already exists across public health agencies and the academic community. However, In practice, public health scientists often operate under more constrained mandates and may have limited capacity to contribute to activities that are not formally supported.

A sustainable model could therefore build on existing distributed expertise through a formalised network of public health and academic partners, supported by modest but dedicated funding. This would enable sustained contributions but retain the responsiveness required for outbreak settings. This network would need a central coordinating body to provide governance, set standards and integrate across academia, the public health sector and existing classification expertise that lies in the ICTV.

Funding models for comparable global surveillance networks often combine public health support and philanthropic sources. Such a system wouldn't require an enormous investment, and the public health impact of this central maintenance could be very high.

We have added a summary of these thoughts to the manuscript, which now reads:
Future consideration should be given to how outbreak nomenclature systems should be supported; at present there are multiple beneficiaries of such systems (e.g. public health agencies, public and private research centres, universities, and industry) but no structure or organisation exists to fund their establishment and maintenance. A sustainable model for lineage system maintenance should leverage existing distributed expertise across public health laboratories and academia, coordinated through a central governance framework, modeled on established pathogen surveillance networks[40,41].

- Occasionally "Pango system," "Pango nomenclature," and "Pango lineage system" are used interchangeably. Consider standardising usage.
We have modified the text to reduce the level of variation of phrase, and have standardised to "Pango lineage system" or "Pango lineage nomenclature system", however have maintained some variation between the two for paragraph flow.

- Some hyphenation inconsistencies (e.g. sustained-human vs sustained human).
We have changed all instances to sustained-human outbreak.

- I suggest expanding the text on the ICTV/virus naming. For example, Pango may be able to influence discussions within ICTV about how fine-scale viral diversity should be named and communicated. This seems like it would be helpful for the virology community compared to the currant system.

We think this is a really important point the reviewer makes. We have previously been in discussions with the ICTV and the broader virus community stakeholders around describing fine-scale viral diversity, including two authors (O'Toole and Colquhoun) contributing in a BV-BRC meeting discussing exactly that. Formally, the ICTV does not take a role in naming diversity below the species rank. However, in practice members of the ICTV are often involved with these decisions as part of community group discussions, so do contribute to these systems as individuals and members of the wider viral-diversity community.

We have clarified some of the text referring to the distinct roles of the ICTV and the virology community around virus naming at different levels, but believe that the community itself should continue to be engaged in discussions around naming as is the current system. We hope that by providing this generalised framework, we can help streamline those discussions and deliver a roadmap for adopting a Pango lineage nomenclature system.

Reviewer #3:
Expertise: Vaccines, emerging viruses

Colquhoun and colleagues highlight the emergence of the Pango lineage nomenclature system and its applicability, generalizability, and implementation for future genomic surveillance for additional viruses. The article is informative but is some places it is written in a field specific manner which can be hard for the interdisciplinary readership of Nat Micro to comprehend. Some suggestions for the authors follow.

We thank the reviewer for their time reviewing this manuscript and for their comments, which have made us consider the broader context of Pango in a way we previously had not.

1. A table or figure highlighting other methods that have been used for virus classification and how they compare to Pango will be useful for the readership.

To address this comment, we have created a table (Supplementary table 1) describing some of the commonly used terms to characterise virus diversity below the species level and have provided some example cases of when those terms have been applied to different virus systems.

2. Can Pango-style nomenclature framework be expanded beyond viruses to bacteria or cancer mutations or mutations associated with genetic disorders and autoimmune diseases? Why yes or no?

In theory, the Pango nomenclature framework could be adapted to any system with hierarchical phylogenetic relationships that needed an extendable, fine-scale naming system.

Our expertise, and the focus of this manuscript, is the application of Pango nomenclature to viruses. Previously, the Pango nomenclature has been applied to SARS-CoV-2 genomic epidemiology for outbreak response and surveillance. The landscape of viral sub-species nomenclature is very diverse and can be confusing at times (see new Supplementary table 1). Recently, other fine-scale nomenclature systems for different viruses have cropped up that emulate the Pango system, but differ subtly but follow confusingly similar rules. This manuscript aims to formalise the generalisation of this system to other viruses to avoid proliferation of many similar systems. Even restricting this formalisation to viruses would be of huge benefit to the research and public health communities.

The principles underlying the Pango nomenclature could be adapted for some bacteria, although challenges may arise with the added complexity of horizontal gene transfer and plasmid tracking. Limiting classification to the core genome may be insufficient for applications like AMR tracking, which in my mind is of greatest concern from a public health point of view for bacterial genomic epidemiology. Similarly, extending the system

to cancer genomics is a really interesting point and may be feasible, however genome size and extensive structural variation may prove challenging in ways that we cannot fully anticipate.

We believe work could be done to develop this framework to extend to these applications, however would require expertise beyond our scope and close consultation with the bacterial and cancer genomics communities. One of the principles we outline in Figure 5 is to assess whether there is a need for the nomenclature before enacting it for a specific use case. If existing bacterial or cancer genomics systems are not currently fit-for-purpose, if the volume of data being produced exceeds that which can be handled through traditional phylogenetic methods, or if communication barriers exist, then perhaps a Pango framework may offer value.

To address this comment, we have added the following to the text:
"It may also be possible to apply the Pango lineage framework to pathogens with larger genomes, such as bacteria, although the added complexity of horizontal gene transfer would need to be carefully considered. In such contexts, public health priorities such as antimicrobial resistance tracking should be central to a bacterial lineage system."

3. How will Pango framework address human-animal and animal-human transmission? Authors mention growth advantage, recombination, immune escape and vaccine evasion among others for SARS-CoV-2, but highlighting the ability to monitor zoonotic and zooanthroponotic transmission and adaptation will strengthen the approach (if the virus evolves between different hosts). SARS-CoV-2 infected several mammalian species, and the evolutionary source or mechanism for various VOCs remain unknown.

The way the Pango lineage system handles zoonosis or reverse zoonosis depends on the use case. For instance, in the case of MPXV Clade Ia, hundreds to possibly thousands of independent zoonotic spillover events occur annually in the DRC. Most of these events are self-limiting and do not lead to sustained human outbreaks. Here, applying Pango to characterise every separate event would not be helpful, as almost every genome sampled may correspond to an independent zoonosis. In such cases the sampled virus diversity primarily reflects the reservoir population, and a stable clade-based nomenclature for this deeper evolutionary diversity is more appropriate, rather than the dynamic, fine-scale lineage framework that the Pango nomenclature would provide. Pango lineages are better applied when a zoonosis leads to a sustained human outbreak.

For host changes between non-human species, such as the Avian flu outbreak in cattle in the USA, a Pango system could be readily applied to capture fine-scale diversity in

cattle. There is nothing necessarily human-specific about Pango lineages, so the system is adaptable to other hosts.

Regarding reverse-zoonosis of SARS-CoV-2, studies have documented multiple spillovers: at least 109 independent spillovers to deer in the USA ([Feng et al., 2023](#)) and at least five spillovers into mink farms in the Netherlands ([Lu et al., 2021](#)). Whether these events are assigned new lineages depends on community surveillance priorities. For SARS-CoV-2, lineages were focused on tracking virus diversity in the human population, informing surveillance efforts to guide outbreak response. As such, we did not characterise a lineage for each of the reverse-zoonosis events. However, if circulation in the deer or mink populations led to a cluster of sequences with mutations enabling them to transmit more effectively in humans, or have higher virulence, we would have then designated a new lineage. For future virus outbreaks, where multi-host dynamics are central to the surveillance needs, the framework can shift focus to include these events as part of lineage assignment.

We have amended the text to allow for some flexibility in what 'signals' a new lineage:
"Depending on the virus and its context, only some of these, or distinct signals, may be relevant."

The text now also reads:
"A Pango lineage system is directly applicable to enzootic pathogens and can be used to characterise and track viral diversity in animals other than humans. In the case of the rabies virus lineage nomenclature, the lineage system broadly captures diversity circulating in dogs, with humans as dead-end hosts[9]. Further, if multi-host dynamics are central to surveillance needs, the framework can include these events as part of lineage designation."

4. If lineages were to be assigned an 'event' like epidemiological event, how does one confidently rule out the possibility of other overlapping but unreported parameters, like evolution in a new host in this new region or region? How does one ensure the accuracy of assigning an event to a node?

For designating a new lineage, an *event* needs to be associated with at least one SNP (within a given genetic background) so that we can pin-point that branch on a phylogeny. If that branch later becomes associated with other information, such as a new host or entry into a new region, the lineage system can be updated to accommodate that new information. Similarly, if new data comes along and that branch/ SNP is no longer associated with an "event" or the evidence is no longer

compelling (e.g. increased transmissibility later turns out to not be the case), the system can be revised and the lineage removed if necessary. The adaptability of the system is one of its greatest strengths!

We have clarified in the text that it's not just new genetic data that can reshape a lineage definition and the next now reads:

"Lineages can be withdrawn from use if, in the light of additional data or new epidemiological information, their designation criteria are no longer met"

5. Is Pango truly only useful in the event of large outbreaks?

Can it improve upon others ways outbreaks are monitored and can it be used to connect different outbreaks over time for the same pathogen? The authors touch upon this but the limitations of why Pango cannot be used or is not particularly useful for slow evolving viruses or for smaller, disconnected outbreaks can be better explained.

We agree with the reviewer that the Pango nomenclature system as we've described it in this manuscript has the potential to be used for various applications, however the primary purpose we have focused on for this framework is to capture the leading edge of viral outbreaks. We have framed the rules and guidance with that application in mind. To help emphasise our rationale for when a Pango lineage system should be enacted and when it may not be the most appropriate tool, we have moved Supplementary Figure 1 to the main text (now Figure 5) to better highlight the decision process. We have also elaborated on some of the points raised (specifically the slow-evolving and small outbreak points) in the caption for Figure 5.

For viruses with limited sampling, clade-based nomenclature, which emphasises broader-scale, stable phylogenetic groups, is generally more appropriate for capturing deep, historical viral diversity. The observed short-term evolutionary processes within a viral outbreak are not necessarily reflected in the deep-ancestry of a phylogeny spanning decades or centuries. For very slow evolving pathogens, epidemiological events may occur faster than detectable genetic changes, meaning distinct lineages on a phylogeny may not correspond well to an epidemiological "event". For distinct outbreaks that are small in scale, traditional phylogenetic methods may be sufficient without the need of a dynamic lineage system.

To further illustrate how a Pango lineage system could be applied to a virus with long-standing diversity in the human population (but with a more complex host network), we have also added a worked example of how lineages for chikungunya virus could be created in the Pango framework (Figure 6, Supplementary Figures 1 & 2, Supplementary Table 2).
